# Cu-catalyzed cross-coupling reactions of vinyl epoxide with organoboron compounds: access to homoallylic alcohols†

**DOI:** 10.1039/c8ra09048c

**Published:** 2018-12-12

**Authors:** Xiao-Yu Lu, Jin-Song Li, Jin-Yu Wang, Shi-Qun Wang, Yue-Ming Li, Yu-Jing Zhu, Ran Zhou, Wen-Jing Ma

**Affiliations:** College of Materials and Chemical Engineering, Chuzhou University Hui Feng Road 1 Chuzhou 239000 P. R. China; School of Chemistry and Chemical Engineering, AnHui University Hefei 230601 China xiaoyulu@mail.ustc.edu.cn

## Abstract

Copper-catalyzed cross-coupling reactions of vinyl epoxide with arylboronates to obtain aryl-substituted homoallylic alcohols are described. The reaction selectivity was different to that of previously reported vinyl epoxide ring-opening reactions using aryl nucleophiles. The reaction proceeded under mild conditions, affording aryl-substituted homoallylic alcohols with high selectivity and in good to excellent yields. The reaction provides convenient access to aryl-substituted homoallylic alcohols from vinyl epoxide

## Abstract 2

Copper-catalyzed cross-coupling reactions of vinyl epoxide with arylboronates to obtain aryl-substituted homoallylic alcohols are described.

## Graphical abstract

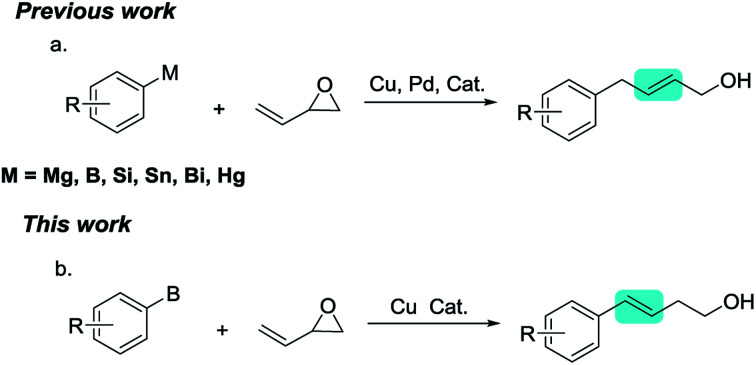

## Introduction

Transition-metal-catalyzed cross-couplings are among the most valuable C–C bond-forming reactions in modern organic synthesis.^1^ Compared with traditional carbon–halogen or carbon–OSO_2_R electrophiles,^2^ epoxides possess ring strain that makes them susceptible to ring opening. Through ring-opening reactions of epoxides, alcohol compounds can be easily obtained with the construction of a C–C bond.^3^ In recent years, various epoxide ring-opening reactions have been reported, including transition-metal-catalyzed Kumada and Negishi-type ring-opening/cross-coupling reactions of epoxides.^4^ Organoboron compounds have excellent stability and are commercially available. Therefore, many groups have reported Suzuki-type ring-opening/coupling reactions of epoxides.^5^ Furthermore, reductive ring-opening/coupling reactions and Heck-type reactions of epoxides have recently been reported.^6^

**Scheme 1:**
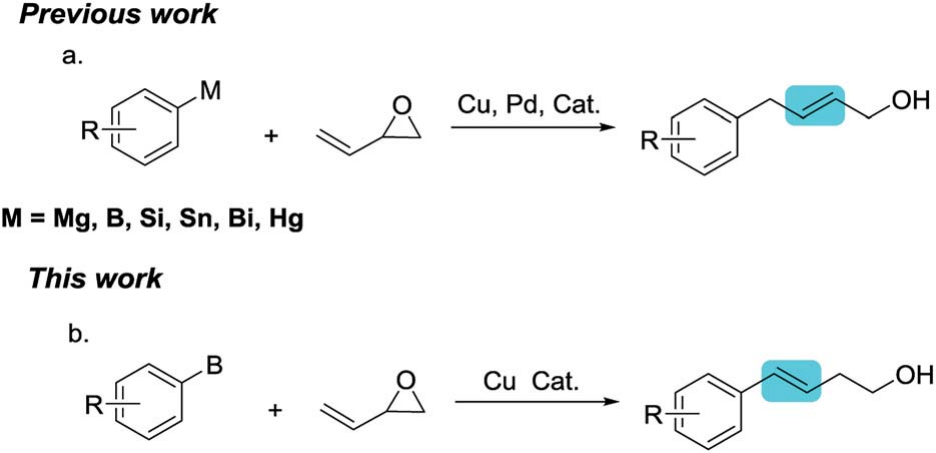
Cross-couplings reactions of vinyl epoxide with aryl vucleophiles.

Vinyl epoxide is a special type of epoxide with rich chemical reactivity. The chemistry of vinyl epoxides is uniquely characterized by the conjugated reactivity of the epoxide and carbon–carbon double bond. Vinyl epoxides are easily prepared using simple synthetic routes and are important building blocks with vast potential in organic synthesis.^7^ Owing to its unique structural features, vinyl epoxide is prone to S_N_2′-type ring-opening/coupling reactions with aryl nucleophiles, affording alcohols containing a C=C bond. Some research groups have reported ring-opening/coupling reactions of vinyl epoxide with various types of aryl nucleophiles, including aryl bismuth compounds,^8^ aryl Grignard reagents,^9^ aryl boron compounds,^10^ aryl siloxane reagents,^11^ and organotin compounds (Scheme 1a). However, these previously reported works all afford aryl-substituted allylic alcohols. Exploring new chemical reaction selectivity is an interesting and challenging area of organic synthesis.

Based on previous work on Cu-catalyzed ring-opening reactions of epoxides with organoboron,^5*c*^ we now report the first example of the Cu-catalyzed ring-opening/cross-coupling of vinyl epoxides with arylboronates, which afforded aryl-substituted homoallylic alcohols (Scheme 1b). The selectivity of the ring-opening reaction was different to that observed previously for ring-opening reactions of vinyl epoxide with aryl nucleophiles. This methodology provides access to synthetically valuable aryl-substituted homoallylic alcohols from vinyl epoxides, which are valuable structural fragments in organic synthesis.

## Experimental

We began our study by selecting *m*-methoxy phenylboronate (1a) and 3,4-epoxy-1-butene (2a) as model reaction substrates (Table 1). We first examined previously reported catalytic conditions for similar Cu-catalyzed ring-opening reactions of epoxides with organoboron compounds.^5*c*,12^ The product was observed, but in low yield (entry 1). Therefore, the previously reported conditions were not suitable for the ring-opening cross-coupling of vinyl epoxide. To assess the roles of diketone, nitrogen, and phosphine ligands, CuI was next evaluated as a copper source (entries 2–6). Using other diketone ligands afforded a lower yield (entries 2 and 3). Using phosphine (entries 4 and 5) and nitrogen (entry 6) ligands instead of diketone ligands increased the reaction yield, but still only obtained a moderate yield. Next, we explored the effect of copper sources on the reaction efficiency (entries 7 and 8). Under the same conditions, using CuCl instead of CuI significantly increased the reaction yield (entries 7 and 8 *vs.* entries 5 and 6). Pleasingly, using CuCl as the copper source resulted in good yields (89% GC yield and 85% isolated yield, entry 8). This might be due to strongly nucleophilic iodide ions affecting the reaction. In ether solvents, hardly any product was obtained (entries 9 and 10). Similarly, almost no product was obtained when using LiOMe as base (entry 11). In the absence of a copper source, no product formation was observed (entry 12).

**Table 1:** Optimization of the reaction conditions

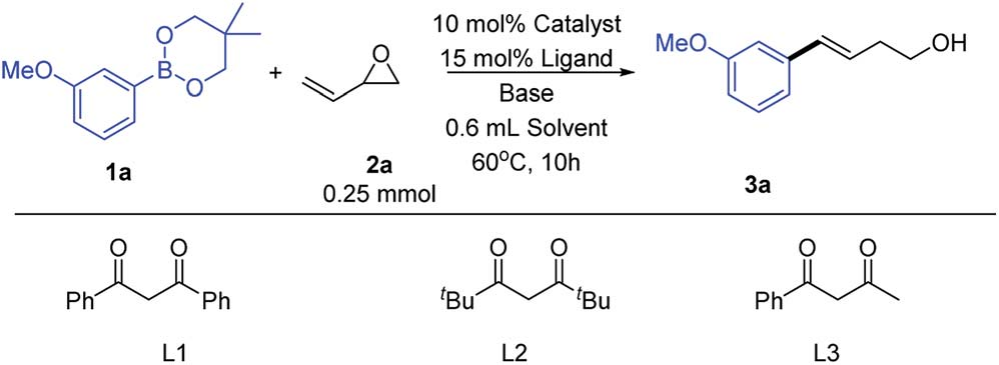					
Entry	Catalyst	Ligand	Base	Solvent	Yield^a^ %
1	CuI	L1	LiO^*t*^Bu	DMF	21
2	CuI	L2	LiO^*t*^Bu	DMF	18
3	CuI	L3	LiO^*t*^Bu	DMF	12
4	CuI	PPh_3_	LiO^*t*^Bu	DMF	15
5	CuI	Xantphos	LiO^*t*^Bu	DMF	41
6	CuI	TMEDA	LiO^*t*^Bu	DMF	52
7	CuCl	Xantphos	LiO^*t*^Bu	DMF	63
**8**	**CuCl**	**TMEDA**	**LiO** ^***t***^ **Bu**	**DMF**	**89**
9	CuCl	TMEDA	LiO^*t*^Bu	THF	Trace
10	CuCl	TMEDA	LiO^*t*^Bu	Dioxane	Trace
11	CuCl	TMEDA	LiOMe	DMF	Trace
12^b^	—	TMEDA	LiO^*t*^Bu	DMF	0

a Reaction conditions: catalyst (10 mol%), base (2.5 equiv.), ligand (15 mol%) in 0.6 mL solvent at 60 °C for 10 h under Ar atmosphere.

b No CuCl. Saturated NH_4_Cl was added after the reaction. The yield was determined by GC using benzophenone as internal standard (average of two GC runs).

With optimized conditions in hand, we next examined the substrate scope (Table 2). To obtain aryl-substituted homoallylic alcohols, it was necessary to add saturated NH_4_Cl after the reaction was complete. Both electron-rich and electron-poor arylboronates afforded good product yields. A series of relevant functional groups, including ether (3a), halogen (3b and 3c), trifluoromethoxy (3d), cyano (3e), and trifluoromethyl (3f) groups, were well tolerated, affording good yields. Notably, aryl halides, including aryl chloride and aryl bromide (3b and 3c), did not hinder the transformation. Therefore, it was possible to perform subsequent cross-coupling reactions at the halogenated positions. A substrate bearing a bicyclic naphthyl group (3g) also participated in the reaction. Unfortunately, alkenylboronate did not participate in the reaction.

**Table 2:** Scope of the reaction^a^

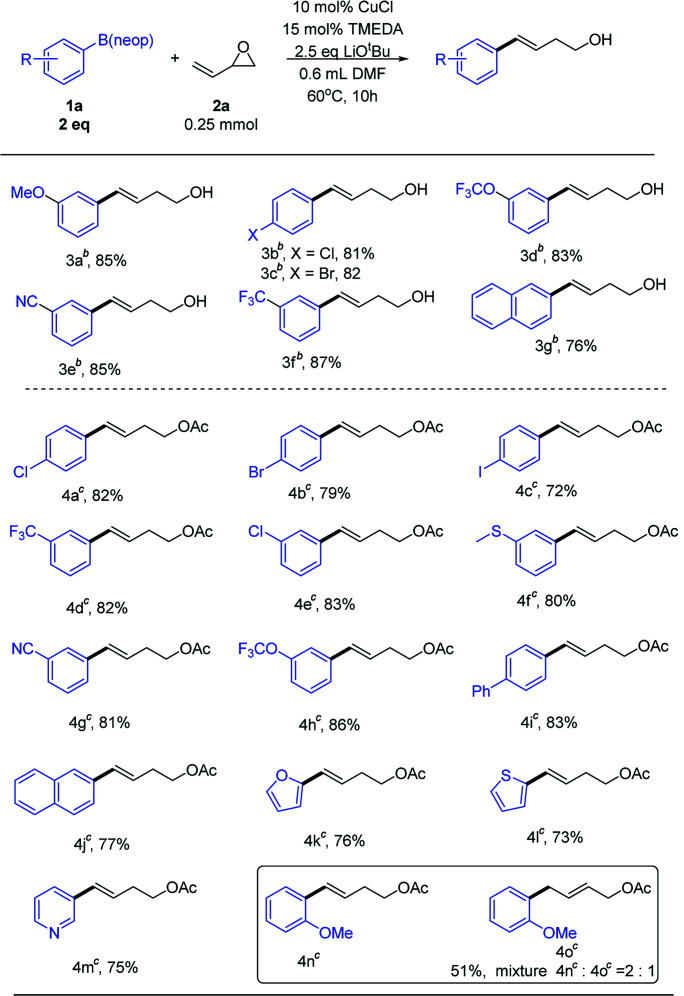

a Reaction conditions: vinyl Epoxides (0.25 mmol), arylboronic esters (2 equiv.), LiO^*t*^Bu (2.5 equiv.).

b Saturated NH_4_Cl was added after the reaction was completed.

c 1–2 mL EtOAc was added after the reaction was completed, then stirred at room temperature for 1–4 h.

Free hydroxyl groups are relatively active functional groups. In many reactions, it is necessary to protect the hydroxyl groups. Hydroxyl groups can be protected with acetyl groups, which requires the use of acetyl chloride or acetic anhydride reagents. Use of these reagents is disadvantageous owing to certain hazards, such as severe irritation and corrosion. After the reaction was completed, then added 1–2 mL EtOAc and stirred at room temperature for 1–4 hour, we obtained acetyl-protected homoallylic (Table 2). Aryl halides, including aryl chloride and aryl bromide (4a and 4b) were also tolerated in this reaction. Notably, the higher activity of aryl iodide (4c) did not affect the transformation. Both electron-rich and electron-poor substrates afforded good yields of acetyl-protected products. A series of relevant functional groups, including trifluoromethyl (4d), halogen (4e), trifluoromethoxy (4h), and cyano (4g) groups, were well tolerated, affording good yields. Arylboronate bearing a sulfur atom (4f) also participated in the reaction. Other aromatic ring substrates, such as biphenyl (4i), naphthyl (4j), furan (4k), thiophene (4l), and pyridine (4m), also participated in the reaction, affording high yields. Steric hindrance at the *ortho* position resulted in a mixture of protected homoallylic and allylic alcohols (4n and 4o). The homoallylic alcohol compound was detected as the only product except for ortho-substituted substrate.^13^

**Scheme 2:**
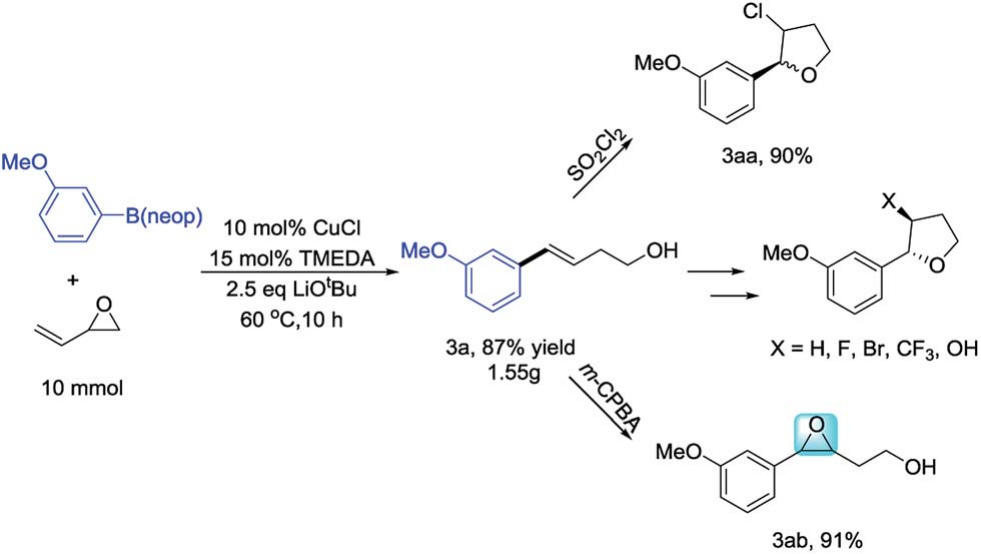
Synthesis of homoallylic alcohbbols *via* ring opening reaction.

To demonstrate the scalability of this method for homoallylic alcohol preparation, we performed the Cu-catalyzed opening-ring reaction of vinyl epoxide on a gram scale, obtaining 3a in 87% yield (Scheme 2). Aryl-substituted homoallylic alcohols are an important organic synthesis fragment, and can be easily converted into β-chlorotetrahydrofurans (3aa). Under chiral catalysis, they can also be converted into chiral β-chlorotetrahydrofurans.^14^ Furthermore, homoallylic alcohols can also be oxidized by *m*-CPBA to 3,4-epoxyalcohols (3ab). 3,4-Epoxyalcohols are important organic synthons that can participate in many reactions.^15^ In addition to these transformations, aryl-substituted homoallylic alcohols can also be converted into β-bromotetrahydrofuran, β-fluorotetrahydrofuran, and β-trifluoromethyl tetrahydrofuran.^16^ Previously, aryl-substituted homoallylic alcohols were generally synthesized through Wittig or alkene metathesis reactions. The Wittig reaction involves a long reaction route, relatively harsh conditions, and requires the use of strong bases. Meanwhile, the reaction can be controlled at low temperature (see, ESI†).^17^ For alkene metathesis, Grubbs catalyst is expensive, while commercial styrene is relatively less (see ESI†).^18^ The Heck reaction of aryl halides with 3-buten-1-ol, generally affords 4-arylbutanal as the major product (see ESI†).^19^ Therefore, to synthesize aryl-substituted homoallylic alcohols, the metal-catalyzed Sonogashira reaction of aryl halides with but-3-yn-1-ol is necessary, followed by alkyne reduction.^20^ These alternative methods clearly show that the reaction reported herein provides an efficient, economical, and convenient method for the synthesis of aryl-substituted homoallylic alcohols.

**Scheme 3:**
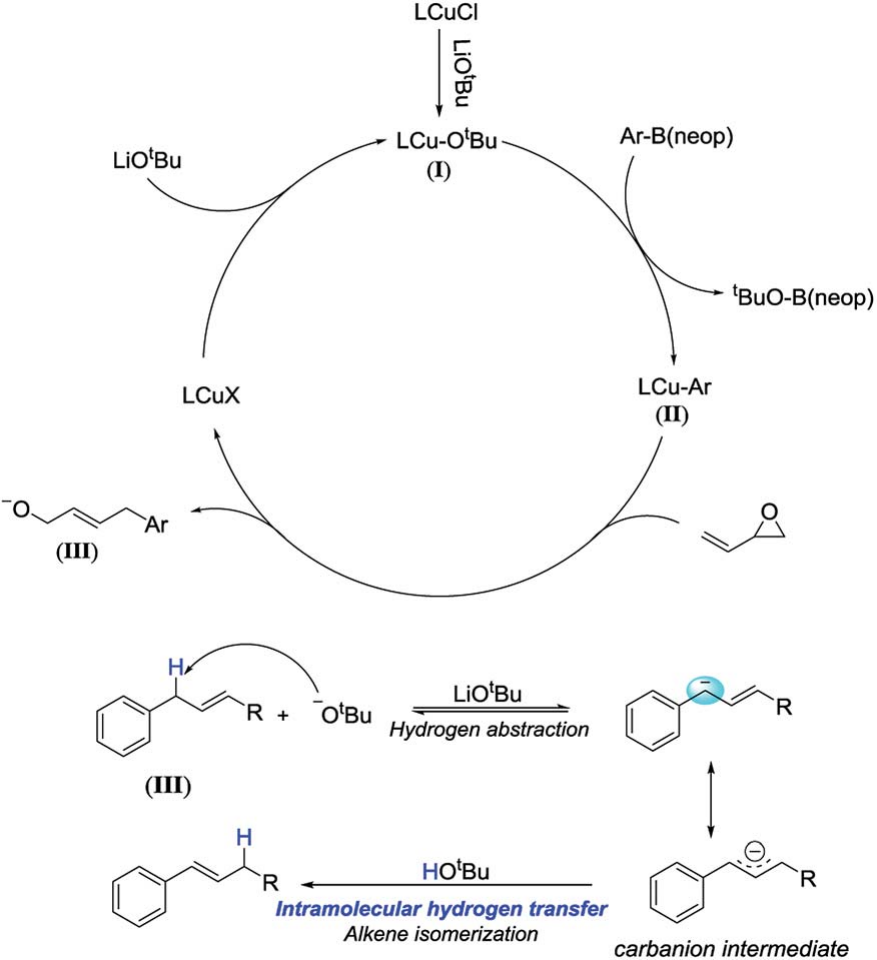
Proposed catalytic cycle.

To illustrate the reaction mechanism, we performed a series of reactions using (*E*)-4-phenylbut-2-en-1-ol (Table 3, eqn (1)). The isomerization product could not be obtained without adding reagents (entry 1). Adding 10 mol% CuCl or 15 mol% TMEDA also did not afford any isomerized product (entries 2 and 3). When 2.5 equiv. of LiO^*t*^Bu was added, the isomerization product was obtained in high yield and no residual material was detected (entry 4). In contrast, adding 2.5 equiv. of LiCl did not afford any isomerization products (entry 5). This confirmed that lithium ions did not cause isomerization. Similarly, the LiO^*t*^Bu can also promote the alkene isomerization of allylbenzene (eqn (2)). It is confirmed by these experiments that the LiO^*t*^Bu is the cause of the alkene migration. Previous studies have indicated that the base, such as K_3_PO_4_, KF, KOH, NaO^*t*^Bu, KO^*t*^Bu,^21^ can promote the alkene isomerization of allylbenzene. Researchers have confirmed that the isomerization goes through an intramolecular base-promoted hydrogen transfer process (carbanion intermediate).^21*a*,21*e*^ From these experiments, we proposed a possible catalytic cycle (Scheme 3). The copper salt first reacts with LiO^*t*^Bu, generating LCu-O^*t*^Bu (I). Transmetalation then produces LCu-Ar(ii), which reacts with vinyl epoxide *via* an S_N_2′-type ring-opening/coupling to afford intermediate (III). Intermediate III then undergoes intramolecular hydrogen transfer and alkene isomerization to afford the aryl-substituted homoallylic alcohols.1
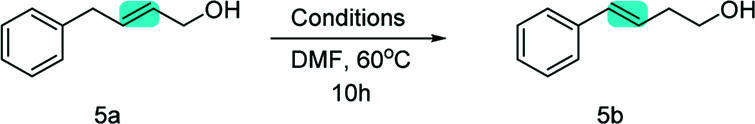
2
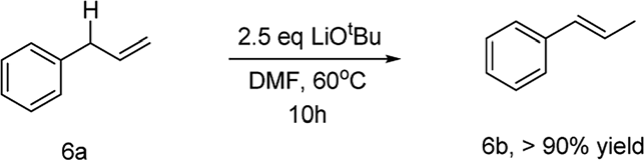


**Table 3:** Support experiments for the proposed mechanism

Entry	Conditions	RSM of 5a	Yield of 5b
1	—	>99%	0
2	10% CuCl	>99%	0
3	15% TMEDA	>99%	0
**4**	**2.5 equiv. LiO** ^***t***^ **Bu**	**trace**	**>95%**
5	2.5 equvi. LiCl	100%	0

## Conclusions

In summary, we have developed the first Cu-catalyzed ring-opening reaction of vinyl epoxides with arylboronates. This reaction affords (*E*)-aryl-substituted homoallylic alcohols. The reaction selectivity was different to that of previous ring-opening reactions of vinyl epoxide with aryl nucleophiles. This reaction provides an effective method for the synthesis of aryl-substituted homoallylic alcohols, which are valuable synthetic intermediates in modern organic synthesis. The mild reaction conditions tolerate a wide variety of functional groups.

## Conflicts of interest

There are no conflicts to declare.

## Supplementary Material

RA-008-C8RA09048C-s001
