# Supplementary material for: Cu-catalyzed cross-coupling reactions of vinyl epoxide with organoboron compounds: access to homoallylic alcohols
Source: RSC Adv. 2018 Dec 12;8(72):41561–5. doi: 10.1039/c8ra09048c (PMC9092012; doi:10.1039/c8ra09048c)
Supplement: RA-008-C8RA09048C-s001 [file RA-008-C8RA09048C-s001.pdf]

Electronic Supplementary Material (ESI) for RSC Advances.
This journal is © The Royal Society of Chemistry 2018

Supporting Information

Cu-Catalyzed Cross-Coupling Reactions of Vinyl Epoxide with Organoboron Compounds ： An Access to Homoallylic Alcohols

Table of Contents

I. General Information S2

a). Materials S2

b). Analytical Methods S2

II. Preparation of Substrates S3

III. General Experimental Procedures, Spectral Data and HPLC Data ... S5

IV. Substrate scope S7

V. NMR Spectra S15

I. General Information

a). Materials

All the reactions were carried out in oven-dried schlenk tubes under argon atmosphere (purity ⩾ 99.999%). Copper(I) iodide was purchased from Sinopharm Chemical Reagent Co., Ltd as a off-white powder and refluxed in THF for further purification. The following chemicals were purchased and used as received: LiOtBu (99.9%, Acros), LiOMe (Acros), Arylboronic Acids (Alfa-Aesa or J&K). Anhydrous DMF (Acros) was stored over 4 Å molecular sieves under an argon atmosphere in a septum-capped bottle.

All the other reagents and solvents mentioned in this text were purchased from commercial sources and used without purification.

b). Analytical Methods

1H-NMR, 13C-NMR and 19F-NMR spectra were recorded on a Bruker Avance 400 spectrometer at ambient temperature in CDCl3 unless otherwise noted; Data for 1H-NMR are reported as follows: chemical shift (δ ppm), multiplicity, integration, and coupling constant (Hz). Data for 13C-NMR are reported in terms of chemical shift (δ ppm), multiplicity, and coupling constant (Hz). Gas chromatographic (GC) analysis was acquired on a Shimadzu GC-2014 Series GC System equipped with a flame-ionization detector. GC-MS analysis was performed on Thermo Scientific AS 3000 Series GC-MS System. HRMS analysis was performed on Finnigan LCQ advantage Max Series MS System. HPLC analysis was performed on Waters-Breeze (2487 Dual Absorbance Detector and 1525 Binary HPLC Pump). Chiralpak IC, AD, AS, KM columns were purchased from Daicel Chemical Industries, LTD. Organic solutions were concentrated under reduced pressure on a Buchi rotary evaporator. Flash column chromatographic purification of products was accomplished using forced-flow chromatography on Silica Gel (200-300 mesh).

II. Preparation of Substrates

Synthesis and characterization of organoboronates

Organoboronates were prepared according to literature procedure [1-5]: Organoboronates were prepared from organoboronic acids and 2,2-dimethyl-1,3-propa-nediol in toluene.

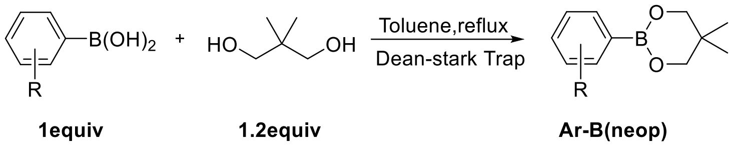

Characterization of organoboronates:

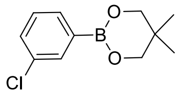

2-(3-chlorophenyl)-5,5-dimethyl-1,3,2-dioxaborinane(CAS: 585524-80-7)
Following general procedure. Purification by silica gel column chromatography gave the product as a white solid. ^{1}H NMR (400 MHz, $CDCl_3$) δ 7.76 (d, *J* = 1.5 Hz, 1H), 7.65 (d, *J* = 7.3 Hz, 1H), 7.39 (m, 1H), 7.28 (t, *J* = 5.9 Hz, 1H), 3.77 (s, 4H), 1.02 (s, 6H).

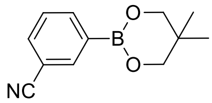

3-(5,5-dimethyl-1,3,2-dioxaborinan-2-yl)benzonitrile (CAS: 214360-45-9)
Following general procedure. Purification by silica gel column chromatography gave the product as a white solid. ^{1}H NMR (400 MHz, $CDCl_3$) δ 8.09 (s, 1H), 8.00 (dd, *J* = 7.5, 1.1 Hz, 1H), 7.69 (m, 1H), 7.45 (t, *J* = 7.6 Hz, 1H), 3.78 (s, 4H), 1.03 (s, 6H).

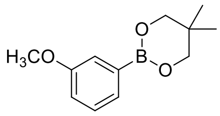

2-(3-methoxyphenyl)-5, 5-dimethyl-1, 3, 2-dioxaborinane (CAS: 1003858-50-1)
Following general procedure. Purification by silica gel column chromatography gave the product as a white solid.^{1}H NMR (400 MHz, $CDCl_3$) δ 7.39 (d, *J*= 7.2 Hz, 1H), 7.33 (d, *J*= 2.6 Hz, 1H), 7.28–7.22 (m, 1H), 7.00–6.92 (m, 1H), 3.82 (s, 3H), 3.77 (s, 4H), 1.02 (s, 6H).^{13}C NMR (101 MHz, $CDCl_3$) δ 159.15, 128.89, 126.37, 118.00, 117.38, 72.44, 55.29, 32.01, 22.03.

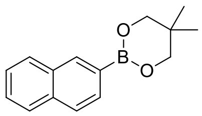

5, 5-dimethyl-2-(naphthalen-2-yl)-1, 3, 2-dioxaborinane (CAS: 627906-96-1)
Following general procedure. Purification by silica gel column chromatography gave the product as a white solid.^{1}H NMR (400 MHz, $CDCl_3$) δ 8.35 (s, 1H), 7.98-7.71 (m, 4H), 7.58 -7.33 (m, 2H), 3.80 (s, 4H), 1.03 (s, 6H). ^{13}C NMR (101 MHz, $CDCl_3$) δ 135.17, 134.99, 133.02, 130.05, 128.80, 127.79, 126.92, 126.75, 125.72, 72.55, 32.09, 22.08.

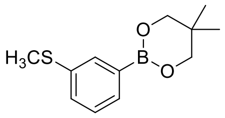

5, 5-dimethyl-2-(3-(methylthio)phenyl)-1,3,2-dioxaborinane

Following general procedure. Purification by silica gel column chromatography gave the product as a white solid. ^{1}H NMR (400 MHz, $CDCl_3$) δ 7.70 (s, 1H), 7.57 (d, *J* = 7.1 Hz, 1H), 7.43–7.26 (m, 2H), 3.77 (s, 4H), 2.50 (s, 3H), 1.03 (s, 6H). ^{13}C NMR (101 MHz, $CDCl_3$) δ 137.65, 132.12, 130.71, 129.22, 128.21, 72.46, 32.03, 22.04, 16.09.

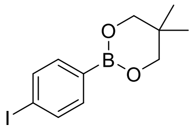

2-(4-iodophenyl)-5,5-dimethyl-1,3,2-dioxaborinane

Following general procedure. Purification by silica gel column chromatography gave the product as a white solid. ^{1}H NMR ($CDCl_3$, 400 MHz) δ 7.70 (d, J =8.1 Hz, 2H), 7.50 (d, J =8.1 Hz, 2H), 3.75 (s, 4H), 1.02 (s, 6H).

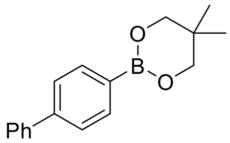

2-([1,1'-biphenyl]-4-yl)-5,5-dimethyl-1,3,2-dioxaborinane

Following general procedure. Purification by silica gel column chromatography gave the product as a white solid. ^{1}H-NMR (400 MHz, $CDCl_3$): δ 7.93-7.88 (m, 2H), 7.68-7.59 (m, 4H), 7.46 (t, J = 7.4 Hz, 2H), 7.37 (t, J = 7.3 Hz, 1H), 3.78 (s, 4H), 1.03 (s, 6H).

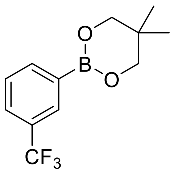

5,5-dimethyl-2-(3-(trifluoromethyl)phenyl)-1,3,2-dioxaborinane (CAS: 635305-32-7)

Following general procedure. Purification by silica gel column chromatography gave the product as a white solid. ^{1}H NMR (400 MHz, $CDCl_3$) δ 8.08 (s, 1H), 7.99 (d, *J* = 7.4 Hz, 1H), 7.67 (d, *J* = 7.8 Hz, 1H), 7.45 (t, *J* = 7.4 Hz, 1H), 3.79 (s, 4H), 1.02 (s, 6H).

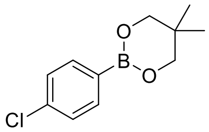

2-(4-chlorophenyl)-5,5-dimethyl-1,3,2-dioxaborinane

Following general procedure. Purification by silica gel column chromatography gave the product as a white solid. ^{1}H-NMR (400 MHz, $CDCl_3$): δ 7.74 (d, J = 8.2 Hz, 2H), 7.33 (d, J = 8.2 Hz, 2H), 3.77 (s, 4H), 1.03 (s, 6H).

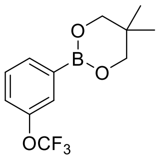

5,5-dimethyl-2-(3-(trifluoromethoxy)phenyl)-1,3,2-dioxaborinane (CAS:635305-40-7)

Following general procedure. Purification by silica gel column chromatography gave the product as a white solid. ^{1}H NMR (400 MHz, $CDCl_3$) δ 7.72 (d, *J* = 7.3 Hz, 1H), 7.64 (s, 1H), 7.38 (t, *J* = 7.7 Hz, 1H), 7.31 – 7.23 (m, 1H), 3.77 (s, 4H), 1.03 (s, 6H)

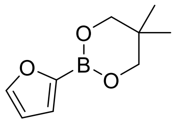

2-(furan-2-yl)-5,5-dimethyl-1,3,2-dioxaborinane (CAS: 941320-88-3)

Following general procedure. Purification by silica gel column chromatography gave the product as a white solid.^{1}H NMR (400 MHz, $CDCl_3$) δ 7.61 (s, 1H), 6.99 (d, *J* = 3.3 Hz, 1H), 6.43 (dd, *J* = 3.1, 1.4 Hz, 1H), 3.76 (s, 4H), 1.02 (s, 6H). ^{13}C NMR (101 MHz, $CDCl_3$) δ 146.65, 121.48, 110.20, 72.29, 32.11, 21.88.

III. General Experimental Procedures, Spectral Data and HPLC Data

Experimental Procedures for Examples Described in Table 1.

In air, Catalyst (10 mmol%), Base (2.5 equiv), Ligand (0.025 mmol) and 2-(3-methoxyphenyl)-5,5-dimethyl-1,3,2-dioxaborinane (2 equiv) were added to a schlenk tube equipped with a stir bar. The vessel was evacuated and filled with argon (three cycles). Solvent (0.5 mL), 2-vinyloxirane (0.25 mmol) were added in turn by syringe. The resulting reaction mixture was stirred vigorously at the mentioned temperature for the indicated amount of time. The reaction was quenched saturated NH_4Cl. The resulting solution was then extracted with CH_2Cl_2 (3 times, 10 mL each), dried over Na_2SO_4, and filtered. Biphenyl was added as internal standard. The product was yielded by GC.

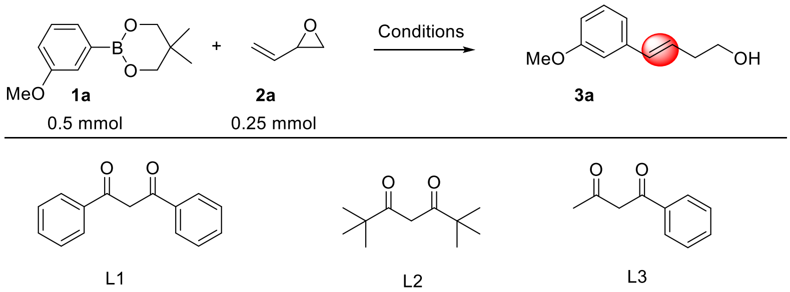

Entry	Catalyst	Ligand	Base	Solvent	T(℃)	Yield [a]
1	CuI	L1	LiO^{t}Bu	DMF	60	21
2	CuI	L2	LiO^{t}Bu	DMF	60	18
3	CuI	L3	LiO^{t}Bu	DMF	60	12

4	CuI	PPh_3	LiO^tBu	DMF	60	15
5	CuI	Xantphos	LiO^tBu	DMF	60	41
6	CuI	TMEDA	LiO^tBu	DMF	60	52
7	CuCl	Xantphos	LiO^tBu	DMF	60	63
**8**	**CuCl**	**TMEDA**	**LiO^tBu**	**DMF**	**60**	**89**
9	CuCl	TMEDA	LiO^tBu	THF	60	trace
10	CuCl	TMEDA	LiO^tBu	dioxane	60	trace
11	CuCl	TMEDA	LiOMe	DMF	60	trace
12	-	TMEDA	LiO^tBu	DMF	60	0

[a]Reaction conditions: 1a (0.5 mmol), 2a (0.25 mmol), Cu salt (10 mol%), Base (2.5 equiv), ligand (15 mol%) in 0.6 mL solvent at 60°C for 10h under Ar atmosphere. The yield was determined by GC (average of two GC runs). DMF=N,N-dimethylformamide.

General procedure A:

In air, CuCl (10 mol%), Li^tOBu (2.5 eq), and arylboronate (2 eq) were added to a schlenk tube equipped with a stir bar. The vessel was evacuated and filled with argon (three cycles). DMF (0.6 mL), vinyl epoxide (0.25 mmol), and TMEDA (15 mol%) were added in turn by syringe. The resulting reaction mixture was stirred vigorously at 60 °C for 10h. The reaction was quenched with saturated NH_4Cl (1-2 mL). The resulting solution was then extracted with CH_2Cl_2 (3 times, 10 mL each), dried over Na_2SO_4, and filtered, concentrated, and purified by column chromatography.

General procedure B:

In air, CuCl (10 mol%), Li^tOBu (2.5 eq), and arylboronate (2 eq) were added to a schlenk tube equipped with a stir bar. The vessel was evacuated and filled with argon (three cycles). DMF (0.6 mL), vinyl epoxide (0.25 mmol), and TMEDA (15 mol%) were added in turn by syringe. The resulting reaction mixture was stirred vigorously at 60 °C for 10h. The reaction was quenched with EtOAc (1-2 mL), stir at room temperature for 1-4 hour. The resulting solution was then extracted with CH_2Cl_2 (3 times, 10 mL each), dried over Na_2SO_4, and filtered, concentrated, and purified by column chromatography.

Experimental Procedures for Examples Described in Scheme 2.

In air, CuCl (10 mol%), Li^tOBu (2.5 eq), and arylboronate (2 eq) were added to a schlenk tube equipped with a stir bar. The vessel was evacuated and filled with argon (three cycles). DMF (20 mL), vinyl epoxide (10 mmol), and TMEDA (15 mol%) were added in turn by syringe. The resulting reaction mixture was stirred vigorously at 60 °C for 10h. The reaction was quenched with saturated NH_4Cl (15 mL). The resulting solution was then extracted with CH_2Cl_2 (3 times, 20 mL

each), dried over Na_2SO_4, and filtered, concentrated, and purified by column chromatography.

References

(1) Ueno, S.; Chatani, N.; Kakiuchi, F. *J. Am. Chem. Soc.* **2007,** *129*, 6098.

(2) Ukai, K.; Aoki, M.; Takaya, J.; Iwasawa, N. *J. Am. Chem. Soc.*, **2006,** *128*, 8706.

(3) Carlson, B.; Phelan, G. D.; Kaminsky, W.; Dalton, L.; Jiang, X.; Liu, S.; Jen, A. K. Y. *J. Am. Chem. Soc.* **2002**, *124*, 14162.

(4) Tobisu, M.; Kita, Y.; Chatani, N. *J. Am. Chem. Soc.,* **2006,** *128*, 8152.

(5) Gribkov,D. V., Pastine,S. J. , Schnürch,M., Sames,D. *J. Am. Chem. Soc.,* **2007**, 129,11750.

IV. Substrate scope

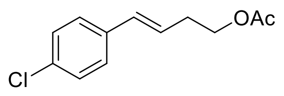

(E)-4-(4-chlorophenyl)but-3-en-1-yl acetate

Following general procedure B, as a pale-yellow liquid. ^{1}H NMR (400 MHz, $CDCl_3$) δ 7.43 – 7.07 (m, 4H), 6.41 (dt, *J* = 15.9, 1.4 Hz, 1H), 6.14 (dt, *J* = 15.9, 7.0 Hz, 1H), 4.18 (t, *J* = 6.7 Hz, 2H), 2.53 (qd, *J* = 6.8, 1.4 Hz, 2H), 2.05 (s, 3H). ^{13}C NMR (101 MHz, $CDCl_3$) δ 171.11, 135.82, 132.92, 131.25, 128.73, 127.37, 126.47, 63.61, 32.40, 21.01. HRMS (APCI) calcd for C12H14ClO2 (M+H+): 225.0677; found: 225.0679.

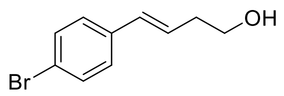

(E)-4-(4-bromophenyl)but-3-en-1-ol

Following general procedure A, as a pale-yellow liquid. ^{1}H NMR (400 MHz, $CDCl_3$) δ 7.50 – 7.34 (m, 2H), 7.24 – 7.09 (m, 2H), 6.41 (d, *J* = 15.9 Hz, 1H), 6.20 (dt, *J* = 15.9, 7.1 Hz, 1H), 3.75 (t, *J* = 6.3 Hz, 2H), 2.46 (qd, *J* = 6.4, 1.3 Hz, 2H), 1.78 (s, 1H). ^{13}C NMR (101 MHz, $CDCl_3$) δ 136.23, 131.61, 131.48, 127.62, 127.40, 120.91, 61.90, 36.36. HRMS (APCI) calcd for C10H11BrNaO (M+Na+): 248.9885; found: 248.9889.

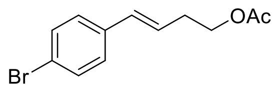

(E)-4-(4-bromophenyl)but-3-en-1-yl acetate

Following general procedure B, as a pale-yellow liquid. ^{1}H NMR (400 MHz, $CDCl_3$) δ 7.51 – 7.33 (m, 2H), 7.23 – 6.87 (m, 2H), 6.40 (d, *J* = 15.9 Hz, 1H), 6.16 (dt, *J* = 15.9, 7.0 Hz, 1H), 4.18 (t, *J* = 6.7 Hz, 2H), 2.53 (qd, *J* = 6.8, 1.4 Hz, 2H), 2.05 (s, 3H). ^{13}C NMR (101 MHz, $CDCl_3$) δ 171.05, 136.19, 131.61, 131.24, 127.64, 126.56, 120.98, 63.51, 32.36, 20.96. HRMS (APCI) calcd for C12H14BrO2 (M+H+): 269.0172; found: 269.0175.

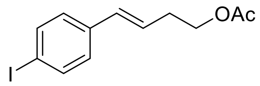

(E)-4-(4-iodophenyl)but-3-en-1-yl acetate

Following general procedure B, as a pale-yellow liquid. ^{1}H NMR (400 MHz, $CDCl_3$) δ 7.85 – 7.38 (m, 2H), 7.14 – 6.98 (m, 2H), 6.38 (d, *J* = 15.9 Hz, 1H), 6.17 (dt, *J* = 15.8, 6.9 Hz, 1H), 4.18 (t, *J* = 6.7 Hz, 2H), 2.53 (qd, *J* = 6.8, 1.3 Hz, 2H), 2.05 (s, 3H). ^{13}C NMR (101 MHz, $CDCl_3$) δ 171.06, 137.58, 136.77, 131.36, 127.90, 126.69, 92.40, 63.50, 32.36, 20.97. HRMS (APCI) calcd for C12H14IO2 (M+H+): 317.0033; found: 317.0035.

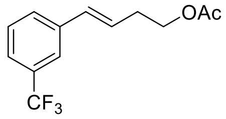

(E)-4-(3-(trifluoromethyl)phenyl)but-3-en-1-yl acetate

Following general procedure B, as a pale-yellow liquid. ^{1}H NMR (400 MHz, $CDCl_3$) δ 7.58 (s, 1H), 7.54 – 7.36 (m, 3H), 6.49 (d, *J* = 15.9 Hz, 1H), 6.26 (dt, *J* = 15.9, 7.0 Hz, 1H), 4.20 (t, *J* = 6.7 Hz, 2H), 2.57 (qd, *J* = 6.8, 1.3 Hz, 2H), 2.06 (s, 3H). ^{19}F NMR (376 MHz, $CDCl_3$) δ -62.79. ^{13}C NMR (101 MHz, $CDCl_3$) δ 171.05, 138.01, 131.08, 130.96 (q, *J* = 36.6 Hz), 129.24, 128.96, 127.84, 126.85 (q, *J* = 272.4 Hz), 123.80 (q, *J* = 3.8 Hz), 122.74 (q, *J* = 3.8 Hz), 63.43, 32.32, 20.91. HRMS (APCI) calcd for C13H14F3O2 (M+H+): 259.0940; found: 259.0943.

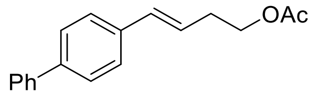

(E)-4-([1,1'-biphenyl]-4-yl)but-3-en-1-yl acetate

Following general procedure B, as a pale-yellow whiod. ^{1}H NMR (400 MHz, $CDCl_3$) δ 7.63

– 7.51 (m, 4H), 7.47 – 7.38 (m, 4H), 7.36 – 7.28 (m, 1H), 6.50 (d, *J* = 15.9 Hz, 1H), 6.21 (dt, *J* = 15.8, 7.0 Hz, 1H), 4.20 (t, *J* = 6.7 Hz, 2H), 2.56 (qd, *J* = 6.8, 1.4 Hz, 2H), 2.06 (s, 3H). ^{13}C NMR (101 MHz, $CDCl_3$) δ 171.13, 140.76, 140.08, 136.34, 131.98, 128.80, 127.28, 127.26, 126.93, 126.54, 125.80, 63.74, 32.46, 21.01. HRMS (APCI) calcd for C18H19O2 (M+H+): 267.1380; found: 267.1386.

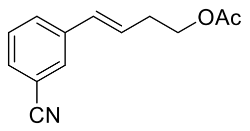

(E)-4-(3-cyanophenyl)but-3-en-1-yl acetate

Following general procedure B, as a pale-yellow liquid. ^{1}H NMR (400 MHz, $CDCl_3$) δ 7.62 (t, *J* = 1.6 Hz, 1H), 7.56 (dt, *J* = 7.8, 1.4 Hz, 1H), 7.50 (dt, *J* = 7.7, 1.4 Hz, 1H), 7.41 (t, *J* = 7.7 Hz, 1H), 6.45 (d, *J* = 15.9 Hz, 1H), 6.26 (dt, *J* = 15.9, 6.9 Hz, 1H), 4.21 (t, *J* = 6.6 Hz, 2H), 2.57 (qd, *J* = 6.7, 1.4 Hz, 2H), 2.07 (s, 3H). ^{13}C NMR (101 MHz, $CDCl_3$) δ 171.01, 138.46, 130.54, 130.27, 129.56, 129.34, 128.82, 118.78, 112.74, 63.29, 32.32, 20.94. HRMS (APCI) calcd for C13H14NO2 (M+H+): 216.1019; found: 216.1025.

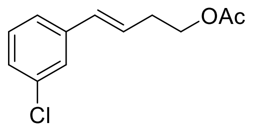

(E)-4-(3-chlorophenyl)but-3-en-1-yl acetate

Following general procedure B, as a pale-yellow liquid. ^{1}H NMR (400 MHz, $CDCl_3$) δ 7.26 (d, *J* = 1.7 Hz, 1H), 7.18 – 7.07 (m, 3H), 6.33 (d, *J* = 15.9 Hz, 1H), 6.11 (dt, *J* = 15.8, 6.9 Hz, 1H), 4.11 (t, *J* = 6.7 Hz, 2H), 2.49 (d, *J* = 1.3 Hz, 2H), 1.98 (s, 3H). ^{13}C NMR (101 MHz, $CDCl_3$) δ 170.03, 138.11, 133.46, 130.09, 128.72, 126.32, 126.20, 125.00, 123.31, 62.47, 31.29, 19.93. HRMS (APCI) calcd for C12H14ClO2 (M+H+): 225.0677; found: 225.0678.

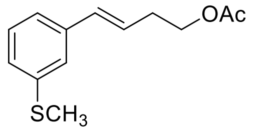

(E)-4-(3-(methylthio)phenyl)but-3-en-1-yl acetate

Following general procedure B, as a pale-yellow liquid. ^{1}H NMR (400 MHz, $CDCl_3$) δ 7.25 – 7.18 (m, 2H), 7.15 – 7.08 (m, 2H), 6.51 – 6.31 (m, 1H), 6.17 (dt, *J* = 15.8, 7.0 Hz, 1H), 4.21 – 4.14 (m, 2H), 2.53 (qd, *J* = 6.8, 1.4 Hz, 2H), 2.48 (s, 3H), 2.05 (s, 3H). ^{13}C NMR (101 MHz,

$CDCl_3$) δ 171.04, 138.69, 131.89, 128.94, 126.39, 125.44, 125.40, 124.34, 122.94, 63.61, 32.34, 20.96, 15.84. HRMS (APCI) calcd for C13H17O2S (M+H+): 237.0944; found: 237.0949.

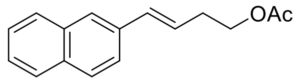

(E)-4-(naphthalen-2-yl)but-3-en-1-yl acetate

Following general procedure B, as a pale-yellow solid. ^{1}H NMR (400 MHz, $CDCl_3$) δ 7.76 (t, *J* = 8.1 Hz, 3H), 7.67 (s, 1H), 7.55 (dd, *J* = 8.6, 1.6 Hz, 1H), 7.48 – 7.36 (m, 2H), 6.60 (d, *J* = 15.9 Hz, 1H), 6.28 (dt, *J* = 15.8, 7.0 Hz, 1H), 4.21 (t, *J* = 6.7 Hz, 2H), 2.57 (qd, *J* = 6.8, 1.3 Hz, 2H), 2.05 (s, 3H). ^{13}C NMR (101 MHz, $CDCl_3$) δ 171.14, 134.75, 133.67, 132.89, 132.54, 128.19, 127.94, 127.68, 126.26, 126.07, 125.82, 125.74, 123.50, 63.76, 32.52, 21.02. HRMS (APCI) calcd for C16H17O2 (M+H+): 241.1223; found: 241.1226.

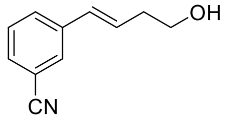

(E)-3-(4-hydroxybut-1-en-1-yl)benzonitrile

Following general procedure A, as a pale-yellow liquid. ^{1}H NMR (400 MHz, $CDCl_3$) δ 7.61 (t, *J* = 1.6 Hz, 1H), 7.56 (dt, *J* = 7.8, 1.4 Hz, 1H), 7.47 (dt, *J* = 7.7, 1.4 Hz, 1H), 7.39 (t, *J* = 7.7 Hz, 1H), 6.46 (d, *J* = 15.9 Hz, 1H), 6.31 (dt, *J* = 15.9, 7.0 Hz, 1H), 3.78 (t, *J* = 6.3 Hz, 2H), 2.51 (qd, *J* = 6.4, 1.3 Hz, 2H), 2.04 (s, 1H). ^{13}C NMR (101 MHz, $CDCl_3$) δ 138.59, 130.42, 130.31, 130.26, 129.86, 129.51, 129.33, 118.85, 112.58, 61.75, 36.26. HRMS (APCI) calcd for C11H11NNaO (M+Na+): 196.0733; found: 196.0738.

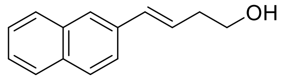

(E)-4-(naphthalen-2-yl)but-3-en-1-ol

Following general procedure A, as a pale-yellow solid. ^{1}H NMR (400 MHz, $CDCl_3$) δ 7.80 – 7.71 (m, 3H), 7.66 (s, 1H), 7.55 (dd, *J* = 8.5, 1.7 Hz, 1H), 7.49 – 7.36 (m, 1H), 6.62 (d, *J* = 15.9 Hz, 1H), 6.30 (dt, *J* = 15.8, 7.1 Hz, 1H), 3.75 (t, *J* = 6.3 Hz, 2H), 2.50 (qd, *J* = 6.4, 1.3 Hz, 2H), 1.84 (s, 1H). ^{13}C NMR (101 MHz, $CDCl_3$) δ 134.78, 133.69, 132.88, 132.82, 128.19, 127.95, 127.69, 126.92, 126.26, 125.80, 125.73, 123.50, 62.09, 36.55. HRMS (APCI) calcd for C14H14NaO (M+Na+): 221.0937; found: 221.0938.

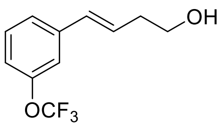

(E)-4-(3-(trifluoromethoxy)phenyl)but-3-en-1-ol

Following general procedure A, as a pale-yellow liquid. ^{1}H NMR (400 MHz, $CDCl_3$) δ 7.27 (ddd, *J* = 9.0, 7.8, 4.6 Hz, 2H), 7.20 (s, 1H), 7.10 – 7.02 (m, 1H), 6.48 (d, *J* = 15.9 Hz, 1H), 6.26 (dt, *J* = 15.9, 7.1 Hz, 1H), 3.78 (t, *J* = 6.3 Hz, 2H), 2.50 (qd, *J* = 6.4, 1.3 Hz, 2H), 1.26 (s, 1H). ^{13}C NMR (101 MHz, $CDCl_3$) δ 149.64, 139.46, 131.36, 129.82, 128.50, 124.54, 120.51 (q, J = 257.0 Hz), 119.51, 118.37, 61.91, 36.31. HRMS (APCI) calcd for C11H11F3NaO2 (M+Na+): 255.0603; found: 255.0605.

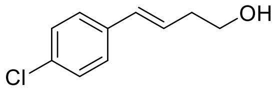

(E)-4-(4-chlorophenyl)but-3-en-1-ol

Following general procedure A, as a pale-yellow liquid. ^{1}H NMR (400 MHz, $CDCl_3$) δ 7.43 – 7.19 (m, 4H), 6.43 (dt, *J* = 15.9, 1.3 Hz, 1H), 6.18 (dt, *J* = 15.9, 7.1 Hz, 1H), 3.75 (t, *J* = 6.3 Hz, 2H), 2.47 (qd, *J* = 6.4, 1.3 Hz, 2H), 1.76 (s, 1H). ^{13}C NMR (101 MHz, $CDCl_3$) δ 135.78, 132.79, 131.45, 128.66, 127.28, 127.23, 61.93, 36.34. HRMS (APCI) calcd for C10H12ClO (M+H+): 183.0571; found: 183.0577.

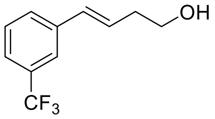

(E)-4-(3-(trifluoromethyl)phenyl)but-3-en-1-ol

Following general procedure A, as a pale-yellow liquid. ^{1}H NMR (400 MHz, $CDCl_3$) δ 7.60 (s, 1H), 7.54 – 7.38 (m, 3H), 6.52 (d, *J* = 15.9 Hz, 1H), 6.31 (dt, *J* = 15.9, 7.1 Hz, 1H), 3.78 (t, *J* = 6.3 Hz, 2H), 2.51 (qd, *J* = 6.4, 1.4 Hz, 2H), 1.67 (s, 1H). ^{19}F NMR (376 MHz, $CDCl_3$) δ -62.78. ^{13}C NMR (101 MHz, $CDCl_3$) δ 138.07, 131.34, 130.96 (q, *J* = 32.1 Hz), 129.29, 128.98, 128.68, 124.17 (q, *J* = 272.3 Hz), 123.77 (q, *J* = 3.8 Hz), 122.71 (q, *J* = 3.8 Hz), 61.92, 36.33. HRMS (APCI) calcd for C10H11ClNaO (M+Na+): 205.0391; found: 205.0396.

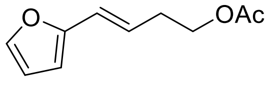

(E)-4-(furan-2-yl)but-3-en-1-yl acetate

Following general procedure B, as a pale-yellow liquid. ^{1}H NMR (400 MHz, $CDCl_3$) δ 7.31

(dd, *J* = 11.2, 9.7 Hz, 1H), 6.55 – 5.94 (m, 4H), 4.16 (t, *J* = 6.7 Hz, 2H), 2.51 (qd, *J* = 6.8, 1.4 Hz, 2H), 2.05 (s, 3H). ^{13}C NMR (101 MHz, $CDCl_3$) δ 171.10, 152.73, 141.63, 124.50, 120.90, 111.16, 106.91, 63.63, 32.10. HRMS (APCI) calcd for C10H13O3 (M+H+): 181.0859; found: 181.0861.

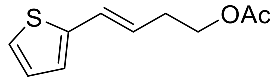

(E)-4-(thiophen-2-yl)but-3-en-1-yl acetate

Following general procedure B, as a pale-yellow liquid. ^{1}H NMR (400 MHz, $CDCl_3$) δ 7.11 (d, *J* = 5.1 Hz, 1H), 6.94 (ddd, *J* = 5.0, 3.5, 1.5 Hz, 1H), 6.90 (d, *J* = 3.2 Hz, 1H), 6.62 – 6.53 (m, 1H), 6.00 (dt, *J* = 15.7, 7.0 Hz, 1H), 4.16 (t, *J* = 6.7 Hz, 2H), 2.50 (qd, *J* = 6.8, 1.5 Hz, 2H), 2.05 (s, 3H). ^{13}C NMR (101 MHz, $CDCl_3$) δ 171.08, 142.36, 127.26, 125.57, 125.42, 124.92, 123.70, 63.56, 32.16, 20.96. HRMS (APCI) calcd for C10H13O2S (M+H+): 197.0631; found: 197.0634.

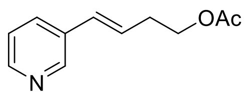

(E)-4-(pyridin-3-yl)but-3-en-1-yl acetate

Following general procedure B, as a pale-yellow liquid. ^{1}H NMR (400 MHz, $CDCl_3$) δ 8.66 – 8.31 (m, 2H), 7.69 (dt, *J* = 8.0, 1.8 Hz, 1H), 7.34 – 7.10 (m, 1H), 6.46 (d, *J* = 16.0 Hz, 1H), 6.27 (dt, *J* = 16.0, 6.9 Hz, 1H), 4.21 (t, *J* = 6.6 Hz, 2H), 2.58 (qd, *J* = 6.7, 1.4 Hz, 2H), 2.07 (s, 3H). ^{13}C NMR (101 MHz, $CDCl_3$) δ 171.06, 148.02, 147.73, 132.93, 128.75, 128.56, 123.57, 63.37, 32.48, 20.96. HRMS (APCI) calcd for C11H14NO2 (M+H+): 192.1019; found: 192.1023.

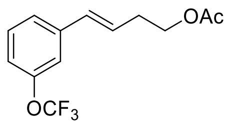

(E)-4-(3-(trifluoromethoxy)phenyl)but-3-en-1-yl acetate

Following general procedure B, as a pale-yellow liquid. ^{1}H NMR (400 MHz, $CDCl_3$) δ 7.35 – 7.23 (m, 2H), 7.19 (s, 1H), 7.11 – 7.02 (m, 1H), 6.45 (d, *J* = 15.9 Hz, 1H), 6.21 (dt, *J* = 15.8, 7.0 Hz, 1H), 4.20 (t, *J* = 6.7 Hz, 2H), 2.56 (qd, *J* = 6.8, 1.3 Hz, 2H), 2.07 (s, 3H). ^{19}F NMR (376 MHz, $CDCl_3$) δ -57.72. ^{13}C NMR (101 MHz, $CDCl_3$) δ 171.12, 149.58, 139.40, 131.05, 129.82, 127.67, 124.52, 120.49 (q, *J* = 257.0 Hz), 119.52, 118.41, 63.46, 32.29, 20.94.

HRMS (APCI) calcd for C13H14F3O3 (M+H+): 275.0890; found: 275.0893.

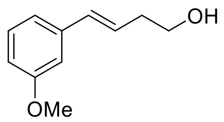

(E)-4-(3-methoxyphenyl)but-3-en-1-ol

Following general procedure A, as a pale-yellow liquid. ^{1}H NMR (400 MHz, $CDCl_3$) δ 7.22 (dd, *J* = 16.0, 8.1 Hz, 1H), 7.01 – 6.84 (m, 2H), 6.76 (m, 1H), 6.46 (d, *J* = 15.8 Hz, 1H), 6.19 (dt, *J* = 15.8, 7.1 Hz, 1H), 3.79 (s, 3H), 3.74 (t, *J* = 6.3 Hz, 2H), 2.47 (qd, *J* = 6.4, 1.4 Hz, 2H), 1.68 (s, 1H). ^{13}C NMR (101 MHz, $CDCl_3$) δ 158.78, 137.70, 131.64, 128.49, 125.71, 117.74, 111.91, 110.39, 60.97, 54.18, 35.34. HRMS (APCI) calcd for C11H14NaO2 (M+Na+):201.0886; found: 201.0888.

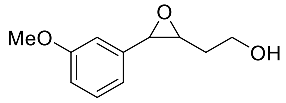

2-(3-(3-methoxyphenyl)oxiran-2-yl)ethan-1-ol

^{1}H NMR (400 MHz, $CDCl_3$) δ 7.24 – 7.2 (m, 1H), 6.86 – 6.81 (m, 2H), 6.77 – 6.76 (m, 1H), 3.82 – 3.80 (m, 2H), 3.76 (s, 3H), 3.69 (d, J= 2.1 Hz, 1H), 3.11 – 3.05 (m, 1H), 2.23 (m, 1H), 2.07 – 2.01 (m, 1 H), 1.86 – 1.79 (m, 1H). ^{13}C NMR (101 MHz, $CDCl_3$) δ 159.9, 139.2, 129.5, 118.1, 114.1, 110.6, 61.1, 59.8, 58.1, 55.3, 34.7.

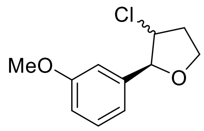

3-chloro-2-(3-methoxyphenyl)tetrahydrofuran

^{1}HNMR (400 MHz, $CDCl_3$) δ 7.19 – 7.23 (m, 1H), 6.89 – 6.82 (m, 2H), 6.76 (m, 1H), 4.93 (d, 1H, J = 4.0 Hz), 4.31 – 4.10 (m, 3H), 3.75 (s, 3H), 2.45 – 2.34 (m, 1H), 2.17 – 2.10 (m, 1H). ^{13}CNMR (101 MHz, $CDCl_3$) δ 159.9, 141.8, 129.6, 117.9, 113.4, 111.1, 88.1, 67.4, 63.3, 55.4, 35.6.

Previous methods of synthesizing aryl-substituted homoallylic alcohols compounds

Methods A. Synthesis of aryl-substituted homoallylic alcohols via wittig reaction.[1]

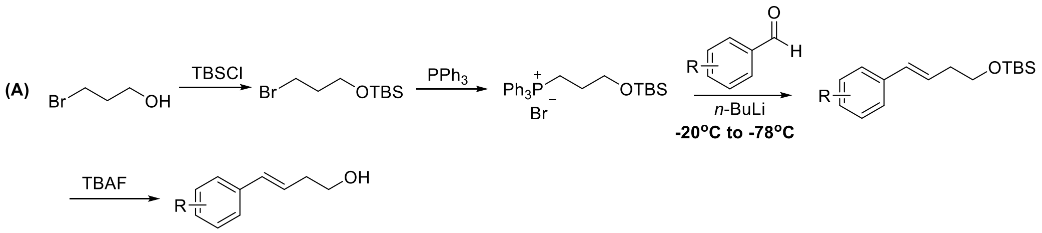

Method B. Synthesis of aryl-substituted homoallylic alcohols via olefin matathesis.[2]

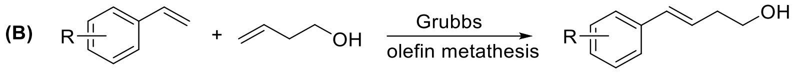

Method C. Heck reaction of aryl halide with 3-buten-1-ol.[3]

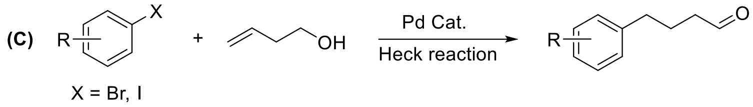

Method D. Synthesis of aryl-substituted homoallylic alcohols via Sonogashira reaction.[4]

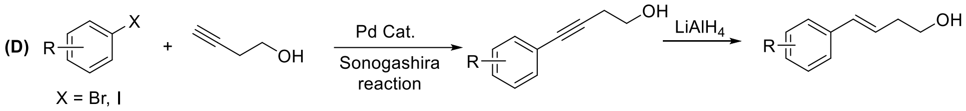

References

[1] (a) B. J. Stokes, S. M. Opra, M. S. Sigman, *J. Am. Chem. Soc.* **2012**, *134*, 11408-11411; (b) A. M. Del Hoyo, A. G. Herraiz, M. G. Suero, *Angew. Chem. Int. Ed.* **2017**, *56*, 1610-1613.

[2] P. R. Walker, C. D. Campbell, A. Suleman, G. Carr, E. A. Anderson, *Angew. Chem. Int. Ed.* **2013**, *52*, 9139-9143.

[3] M. van Gemmeren, M. Borjesson, A. Tortajada, S. Z. Sun, K. Okura, R. Martin, *Angew. Chem. Int. Ed.* **2017**, *56*, 6558-6562.

[4] (a) X. Su, Y. Sun, J. Yao, H. Chen, C. Chen, *Chem. Commun.* **2016**, *52*, 4537-4540; (b) J. Tummatorn, S. Ruchirawat, P. Ploypradith, *Chem. Eur. J.* **2010**, *16*, 1445-1448.

V. NMR Spectra

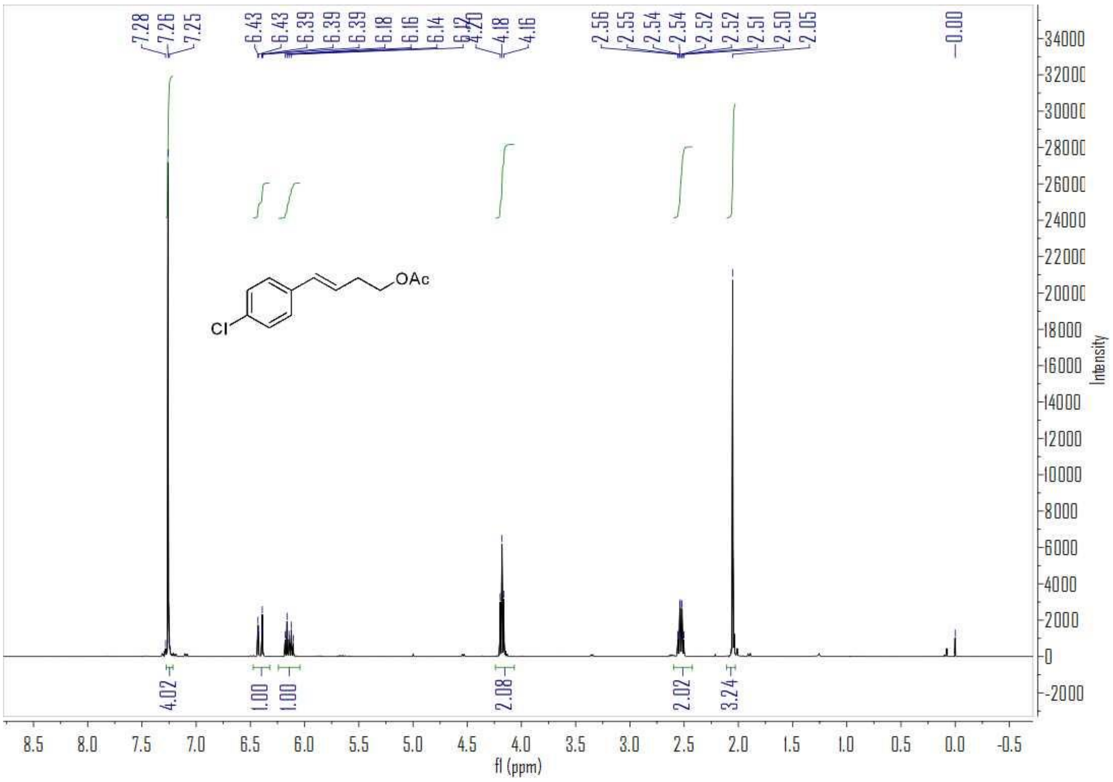

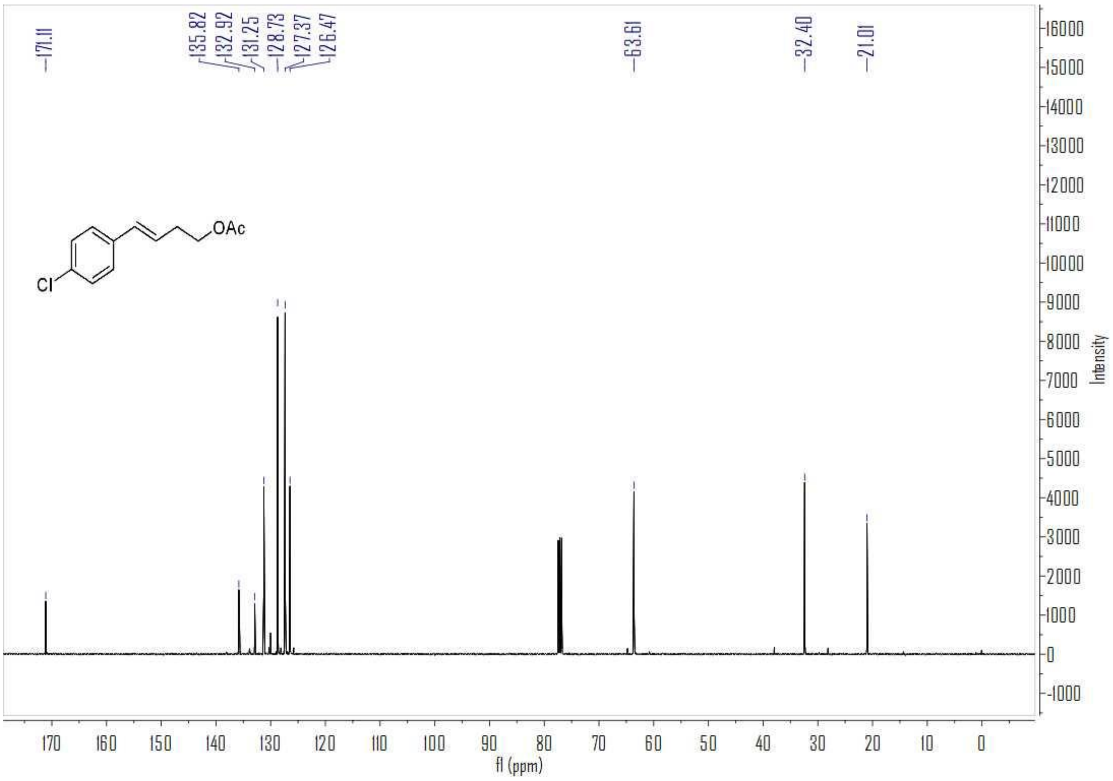

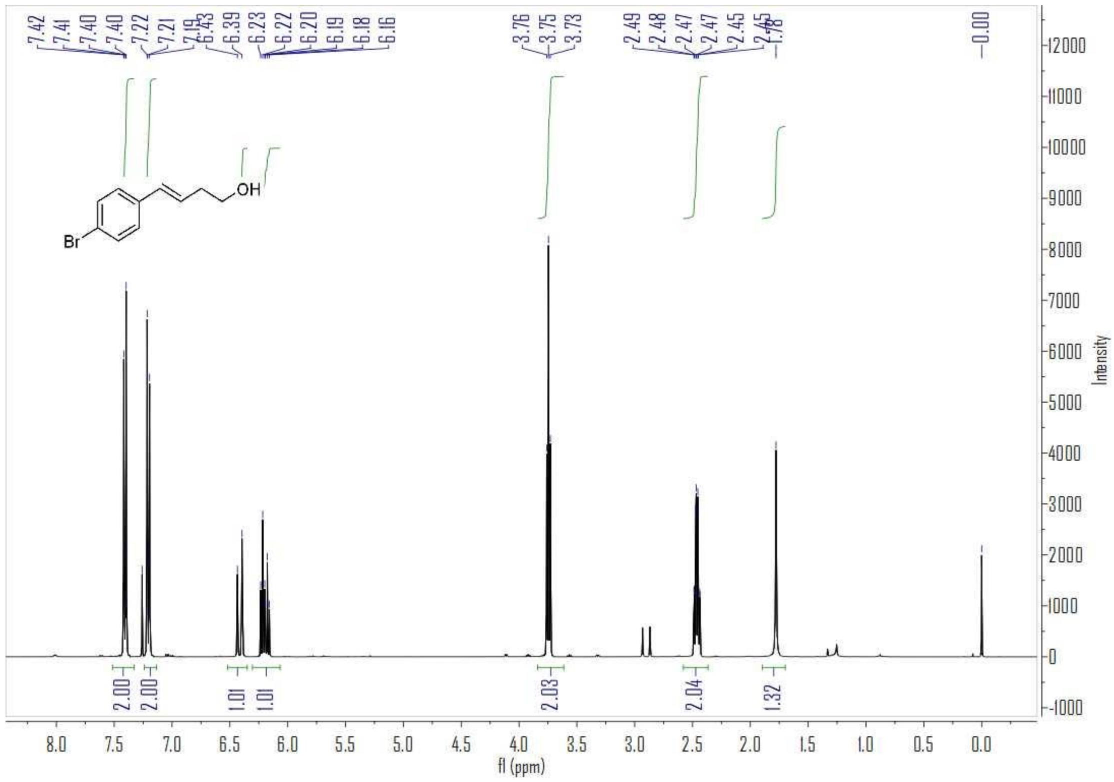
7.42
7.41
7.40
7.40
7.22
7.21
7.19
6.43
6.39
6.23
6.22
6.20
6.19
6.18
6.16
3.76
3.75
3.73
2.49
2.48
2.47
2.47
2.45
1.78
0.00
OH
Br
2.00
2.00
1.01
1.01
2.03
2.04
1.32
fl (ppm)
Intensity

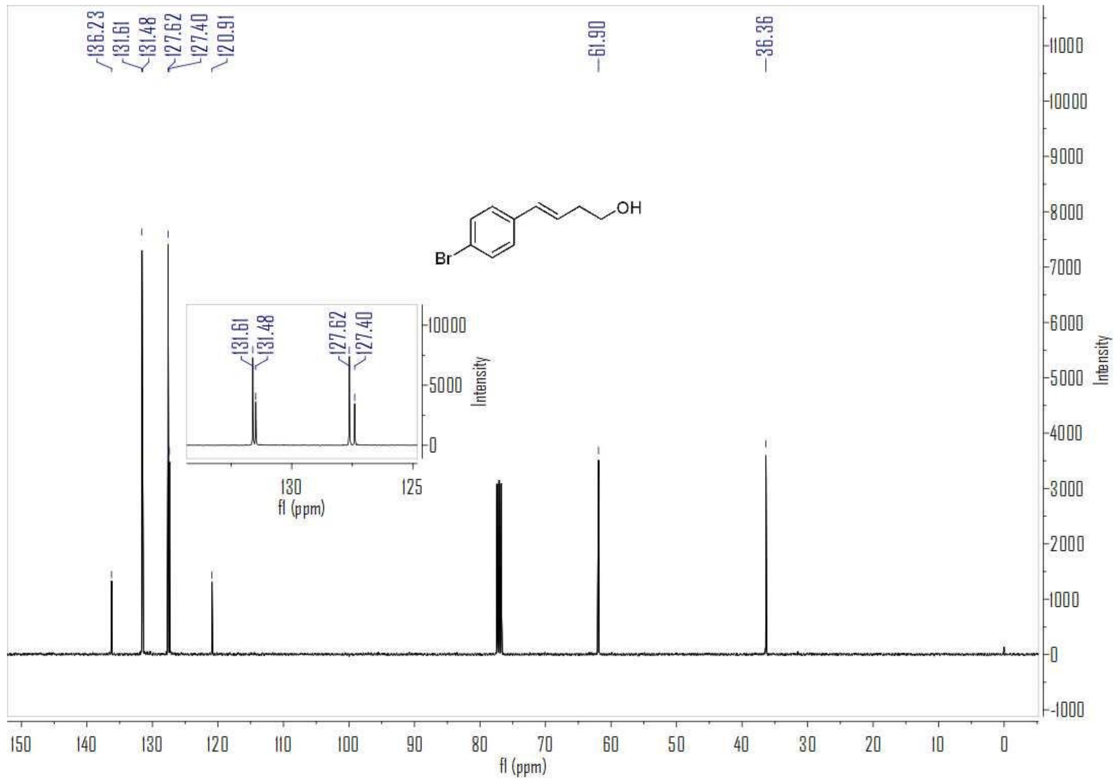
136.23
131.61
131.48
127.62
127.40
120.91
61.90
36.36
OH
Br
131.61
131.48
127.62
127.40
fl (ppm)
Intensity

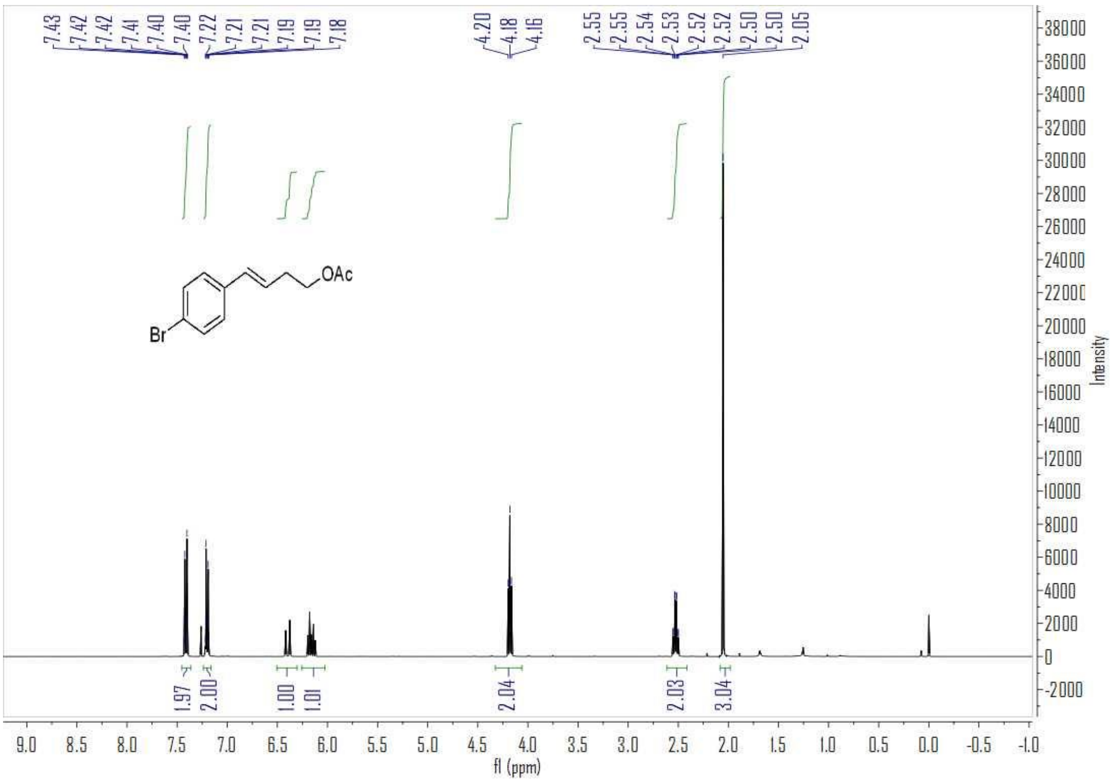
7.43
7.42
7.42
7.41
7.40
7.40
7.22
7.21
7.21
7.19
7.19
7.18
4.20
4.18
4.16
2.55
2.55
2.54
2.53
2.52
2.52
2.50
2.50
2.05
OAc
Br
1.97
2.00
1.00
1.01
2.04
2.03
3.04
f1 (ppm)
Intensity

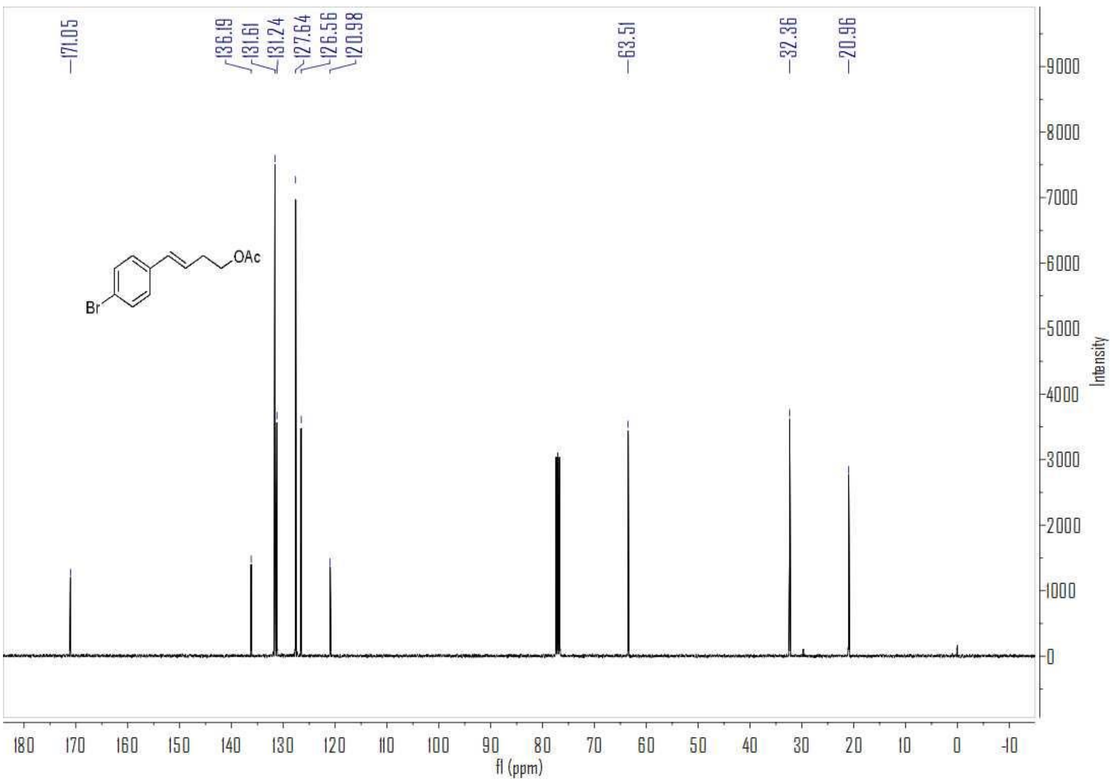
171.05
136.19
131.61
131.24
127.64
126.56
120.98
63.51
32.36
20.96
OAc
Br
f1 (ppm)
Intensity

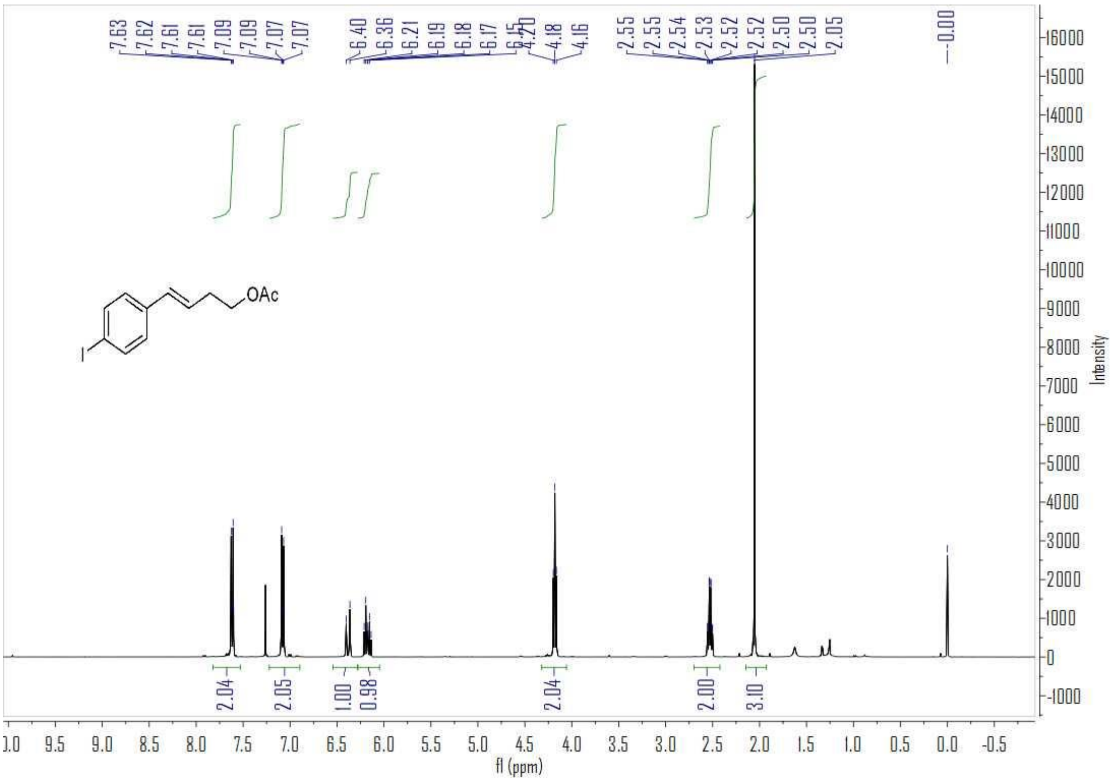
7.63
7.62
7.61
7.61
7.09
7.09
7.07
7.07
6.40
6.36
6.21
6.19
6.18
6.17
6.15
4.20
4.18
4.16
2.55
2.55
2.54
2.53
2.52
2.52
2.50
2.50
2.05
0.00
OAc
I
2.04
2.05
1.00
0.98
2.04
2.00
3.10
fl (ppm)
Intensity

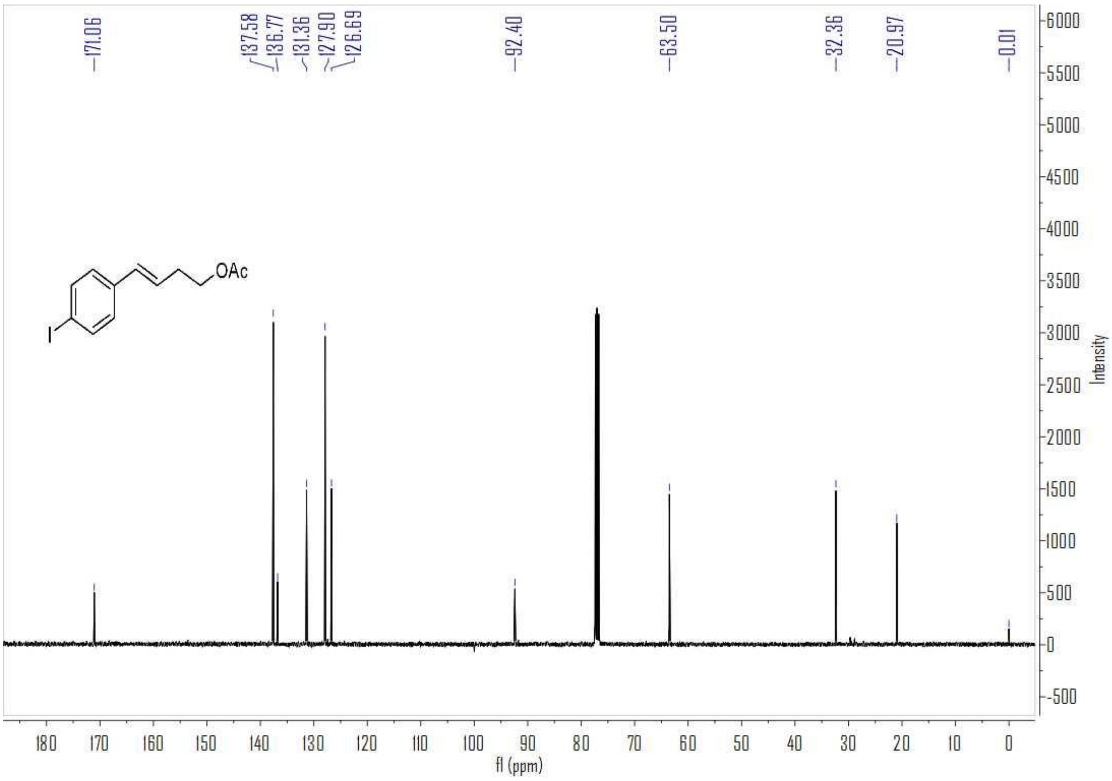
171.06
137.58
136.77
131.36
127.90
126.69
92.40
63.50
32.36
20.97
0.01
OAc
I
fl (ppm)
Intensity

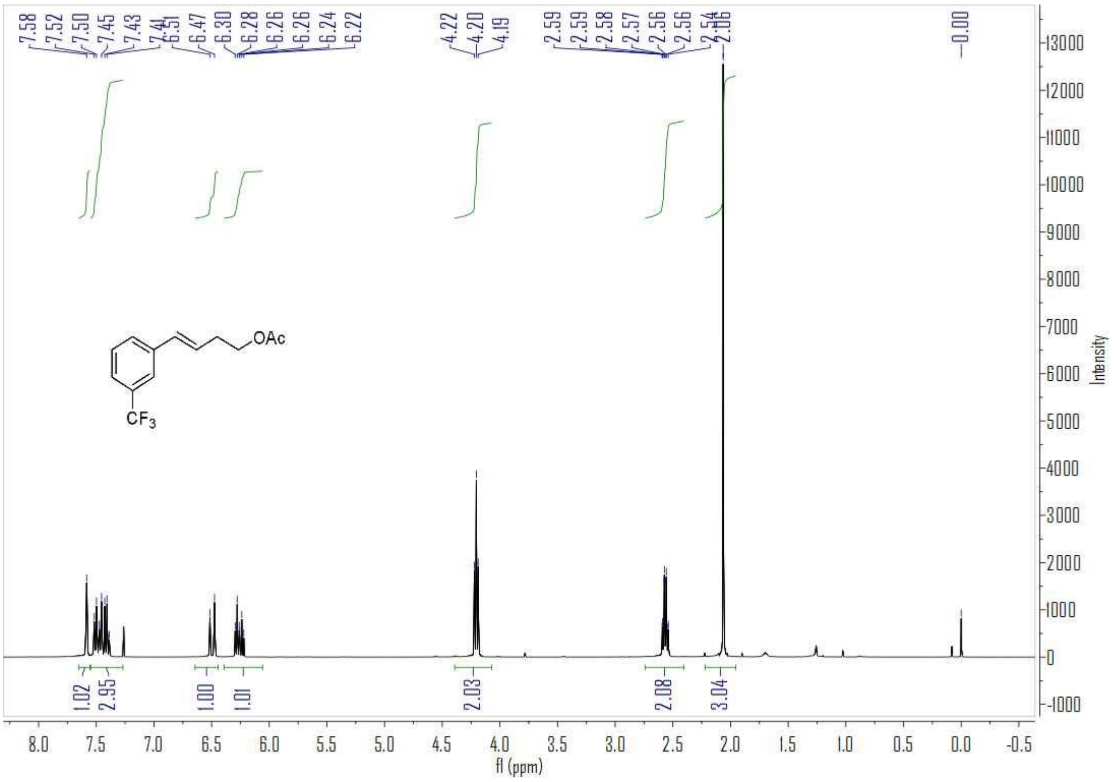

7.58
7.52
7.50
7.45
7.43
7.41
6.51
6.47
6.30
6.28
6.26
6.26
6.24
6.22
4.22
4.20
4.19
2.59
2.59
2.58
2.57
2.56
2.56
2.54
2.06
0.00
OAc
CF3
1.02
2.95
1.00
1.01
2.03
2.08
3.04
fl (ppm)
Intensity

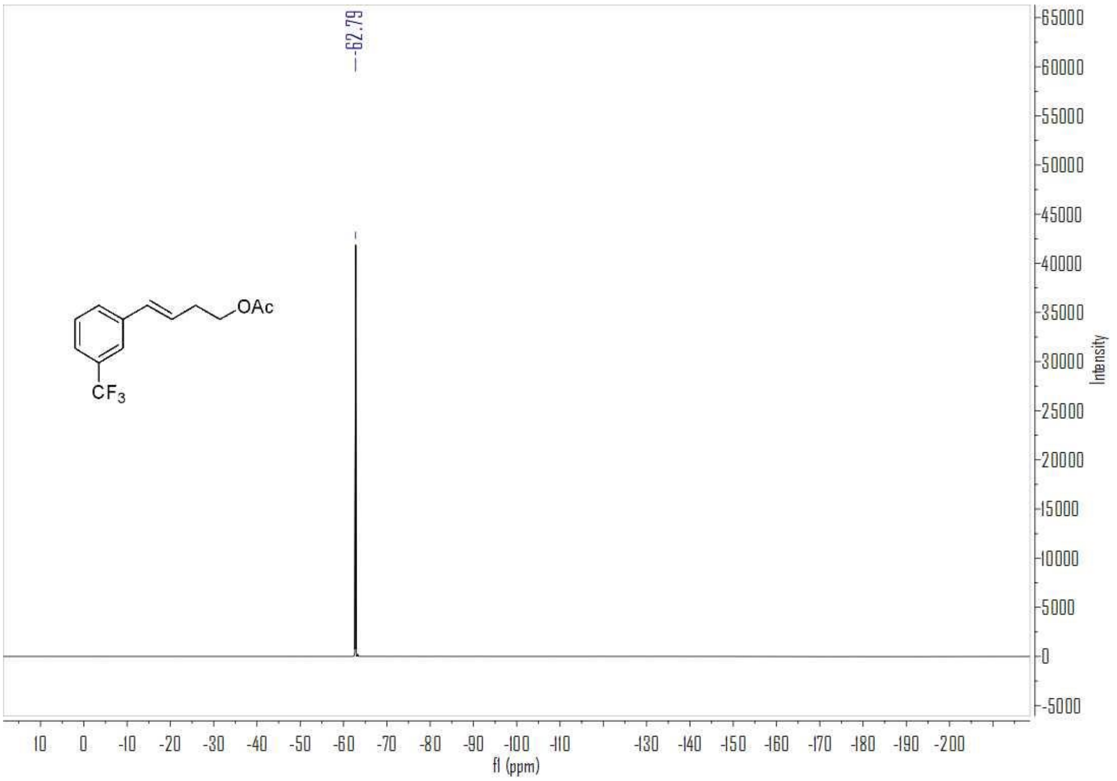

-62.79
OAc
CF3
fl (ppm)
Intensity

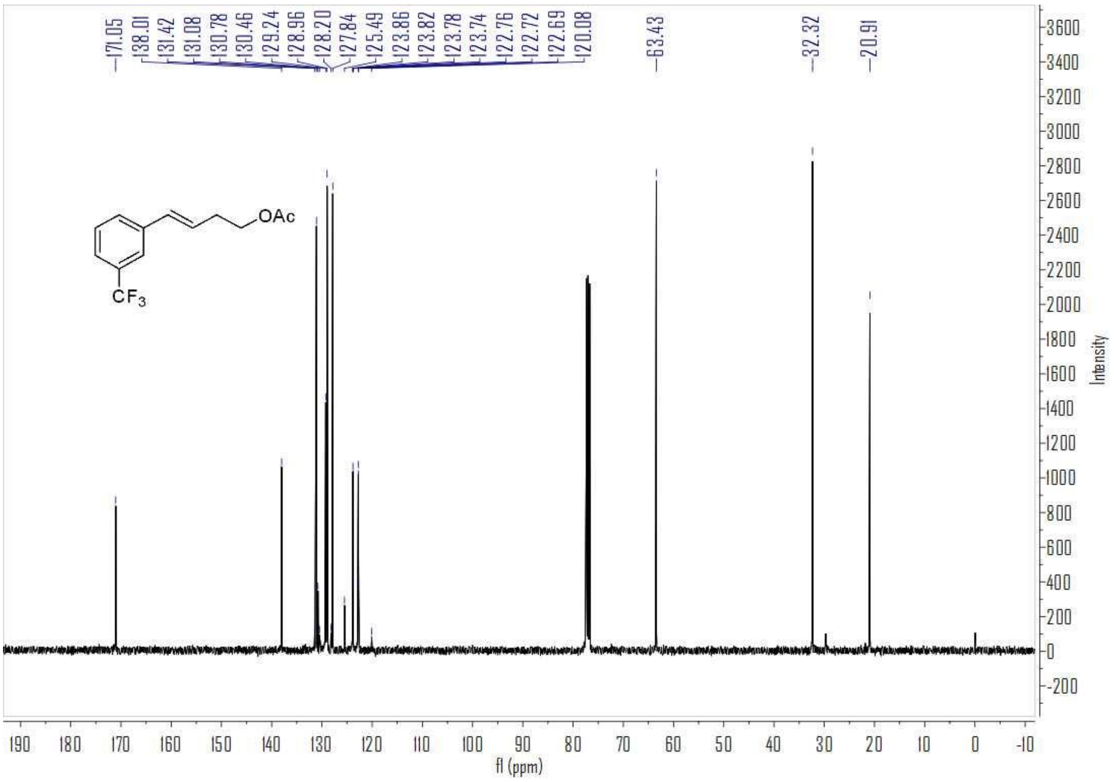

171.05
138.01
131.42
131.08
130.78
130.46
129.24
128.96
128.20
127.84
125.49
123.86
123.82
123.78
123.74
122.76
122.72
122.69
120.08
63.43
32.32
20.91
OAc
CF3
Intensity
f1 (ppm)

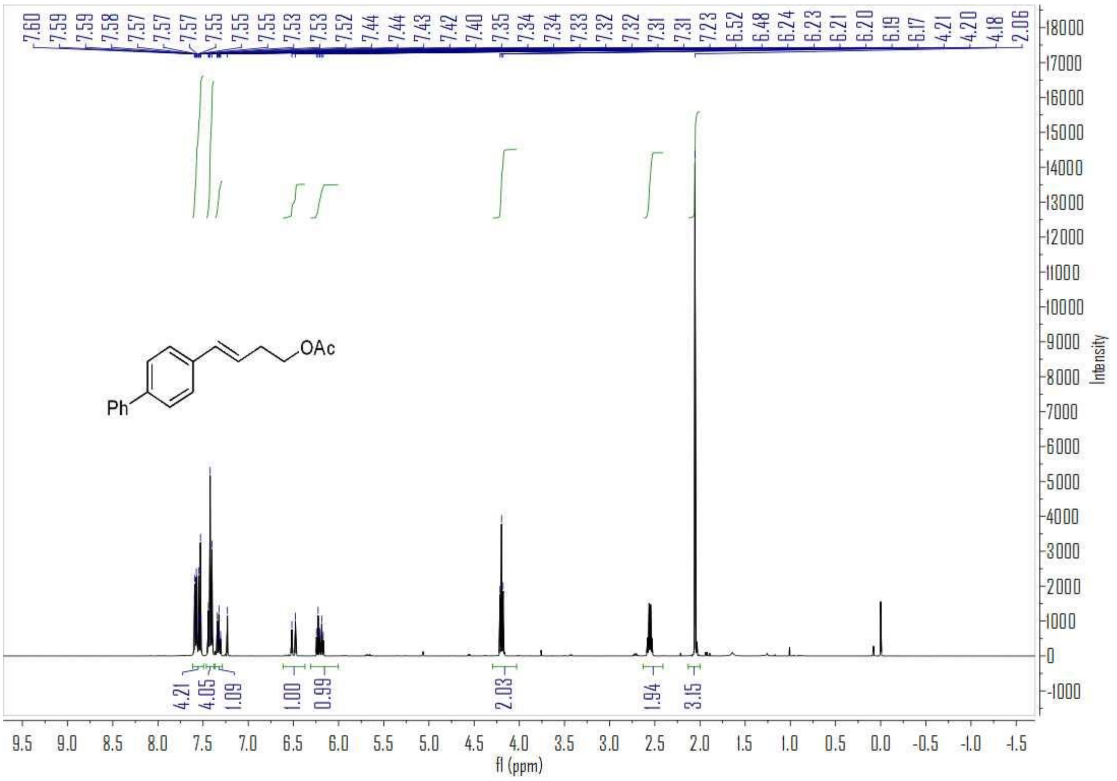

7.60
7.59
7.59
7.58
7.57
7.57
7.57
7.55
7.55
7.55
7.53
7.53
7.52
7.44
7.44
7.43
7.42
7.40
7.35
7.34
7.34
7.33
7.32
7.32
7.31
7.31
7.23
6.52
6.48
6.24
6.23
6.21
6.20
6.19
6.17
4.21
4.20
4.18
2.06
OAc
Ph
4.21
4.05
1.09
1.00
0.99
2.03
1.94
3.15
Intensity
f1 (ppm)

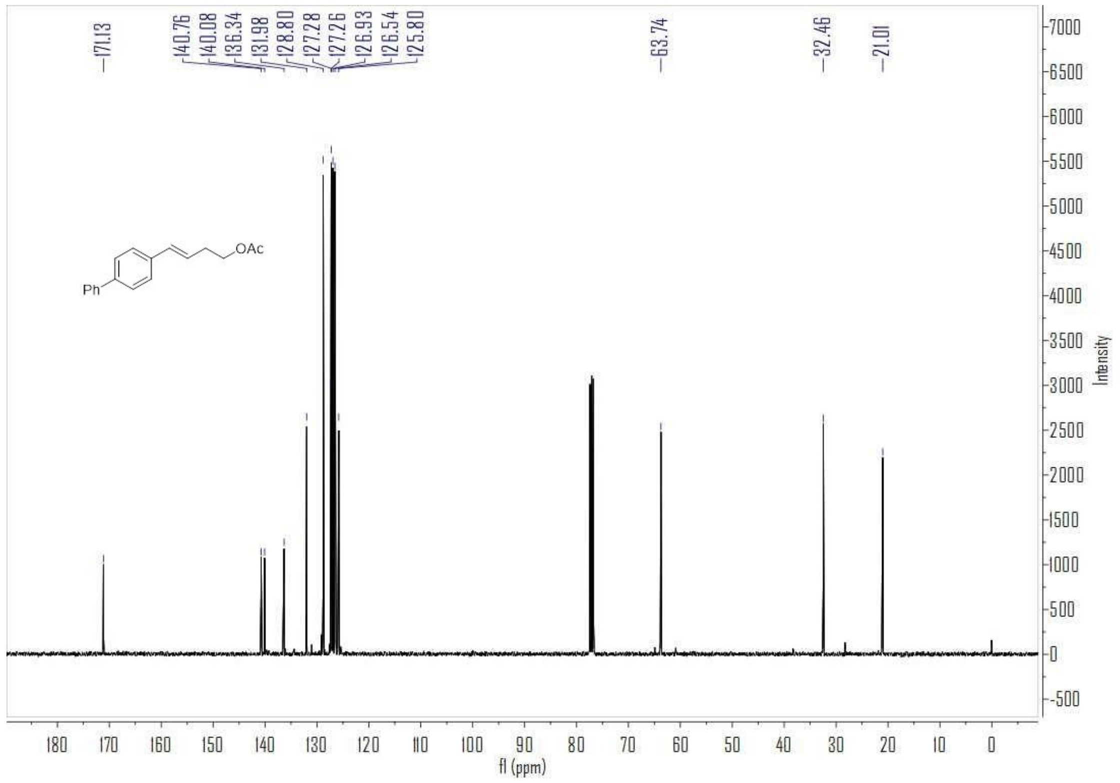

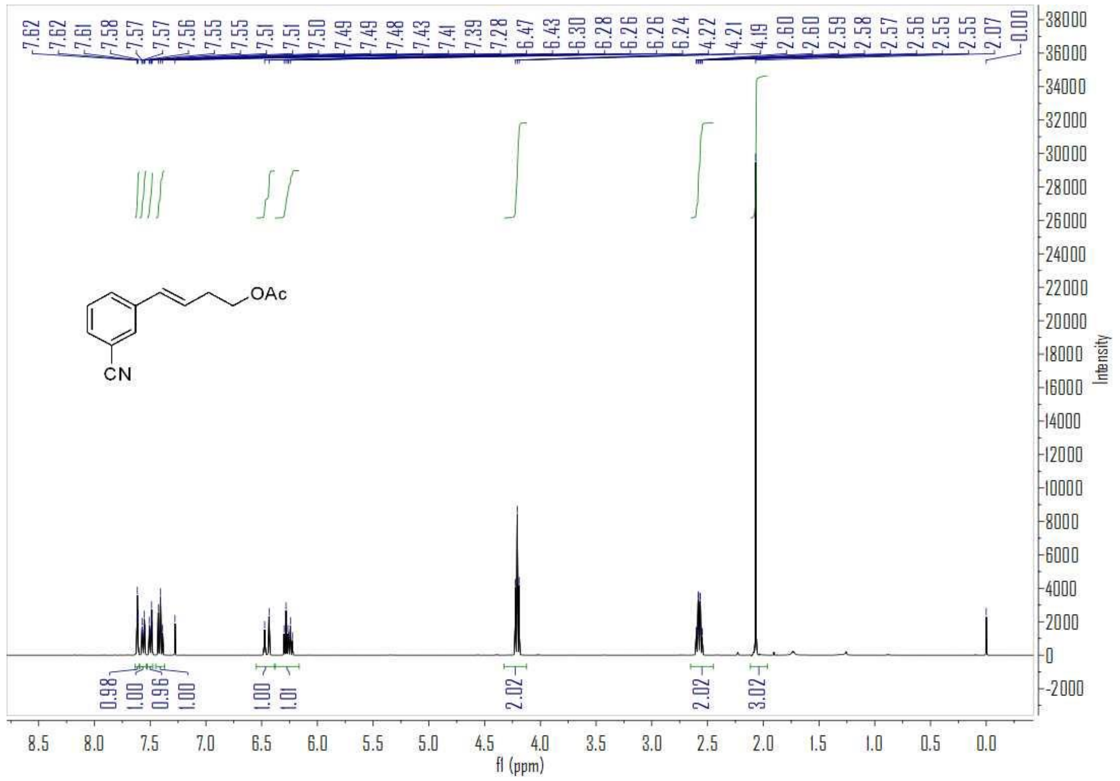

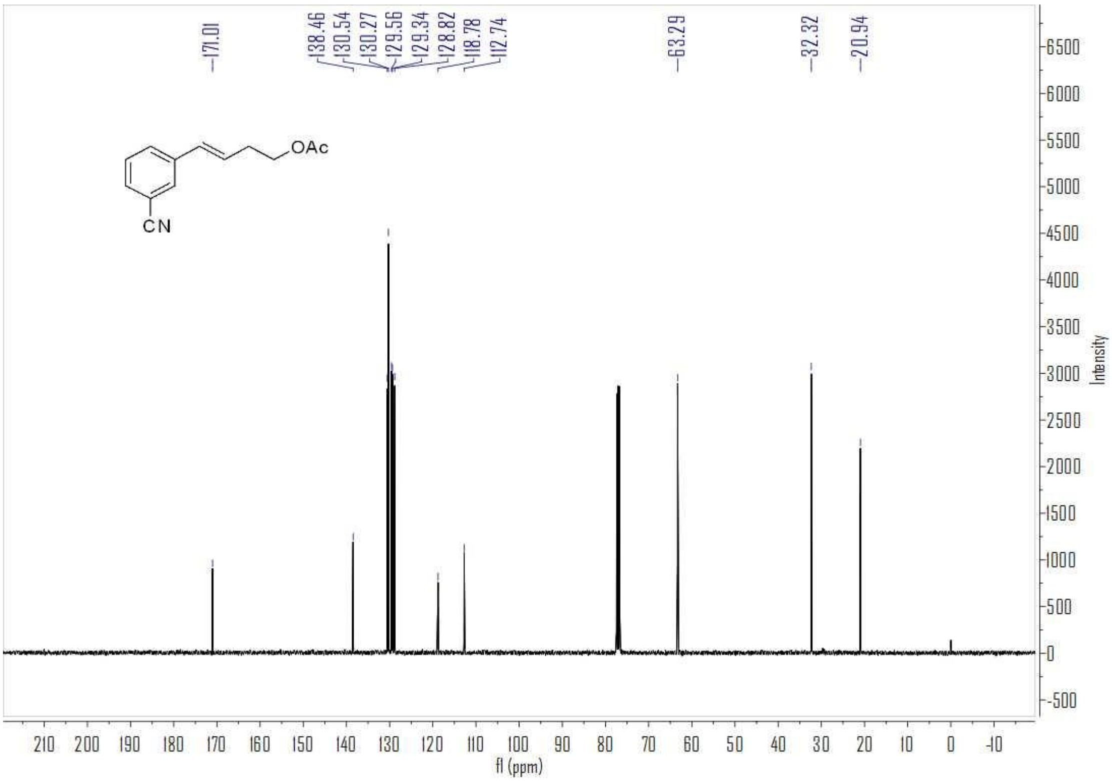
171.01
138.46
130.54
130.27
129.56
129.34
128.82
118.78
112.74
63.29
32.32
20.94
OAc
CN
Intensity
fl (ppm)

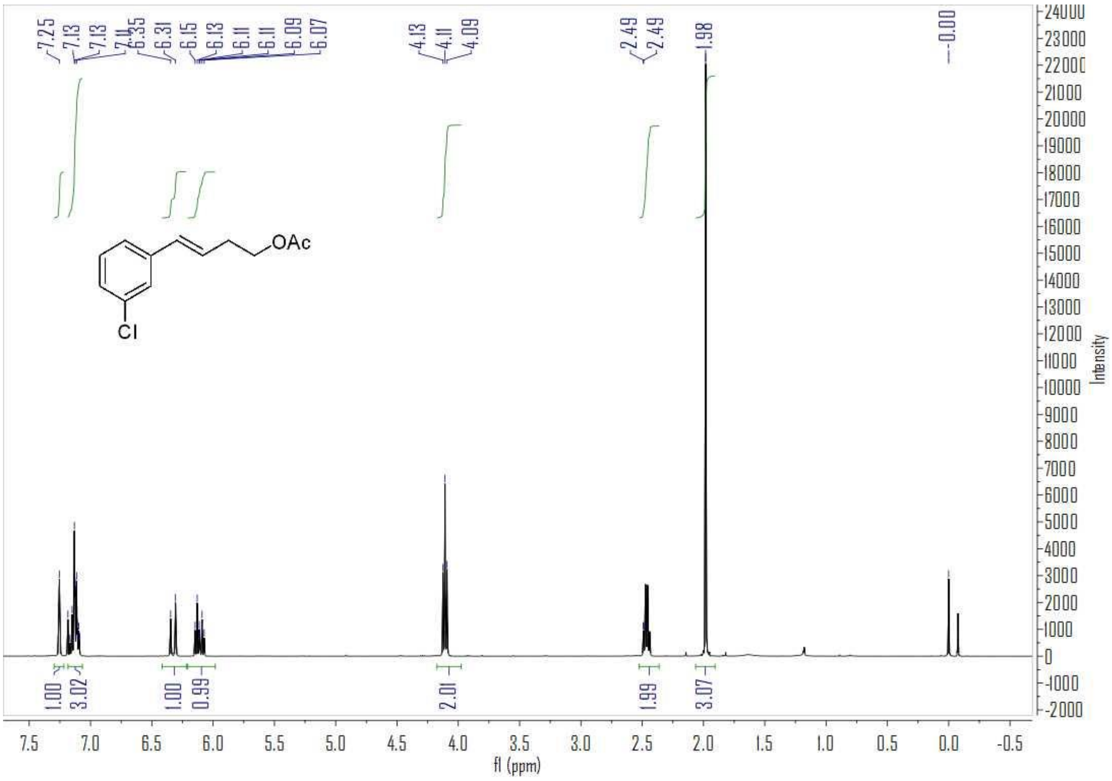
7.25
7.13
7.13
7.11
6.35
6.31
6.15
6.13
6.11
6.11
6.09
6.07
4.13
4.11
4.09
2.49
2.49
1.98
0.00
OAc
Cl
1.00
3.02
1.00
0.99
2.01
1.99
3.07
Intensity
fl (ppm)

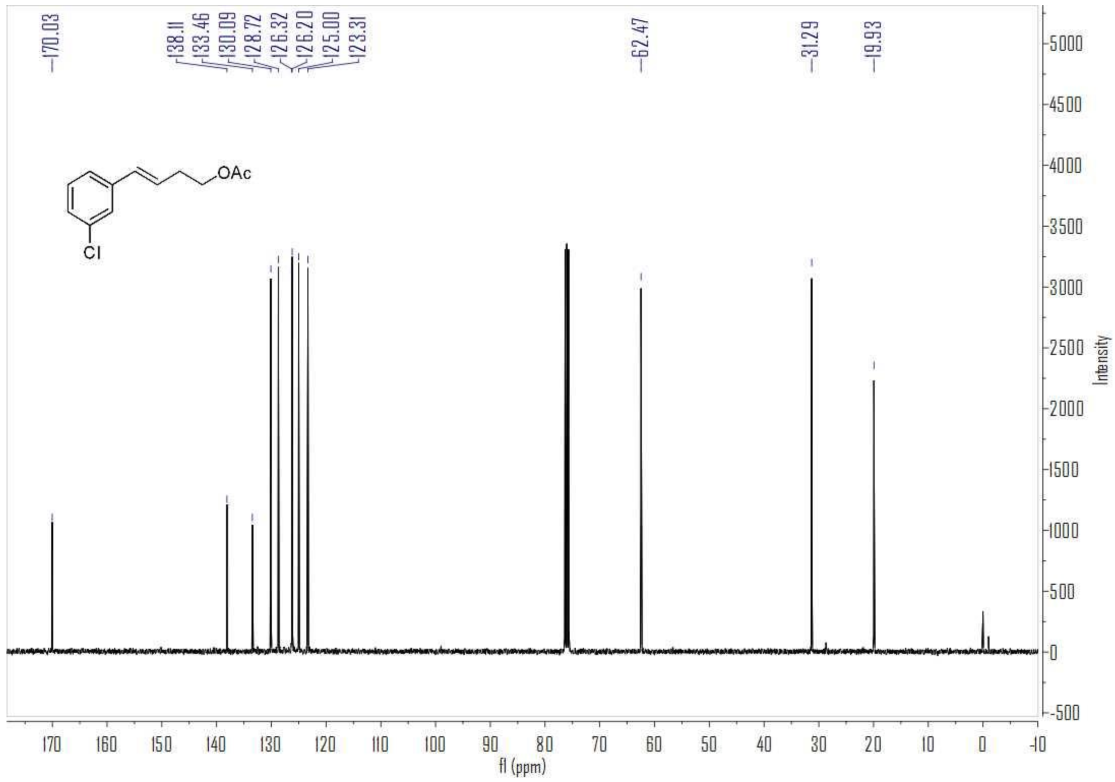

170.03
138.11
133.46
130.09
128.72
126.32
126.20
125.00
123.31
62.47
31.29
19.93
OAc
Cl
Intensity
fl (ppm)

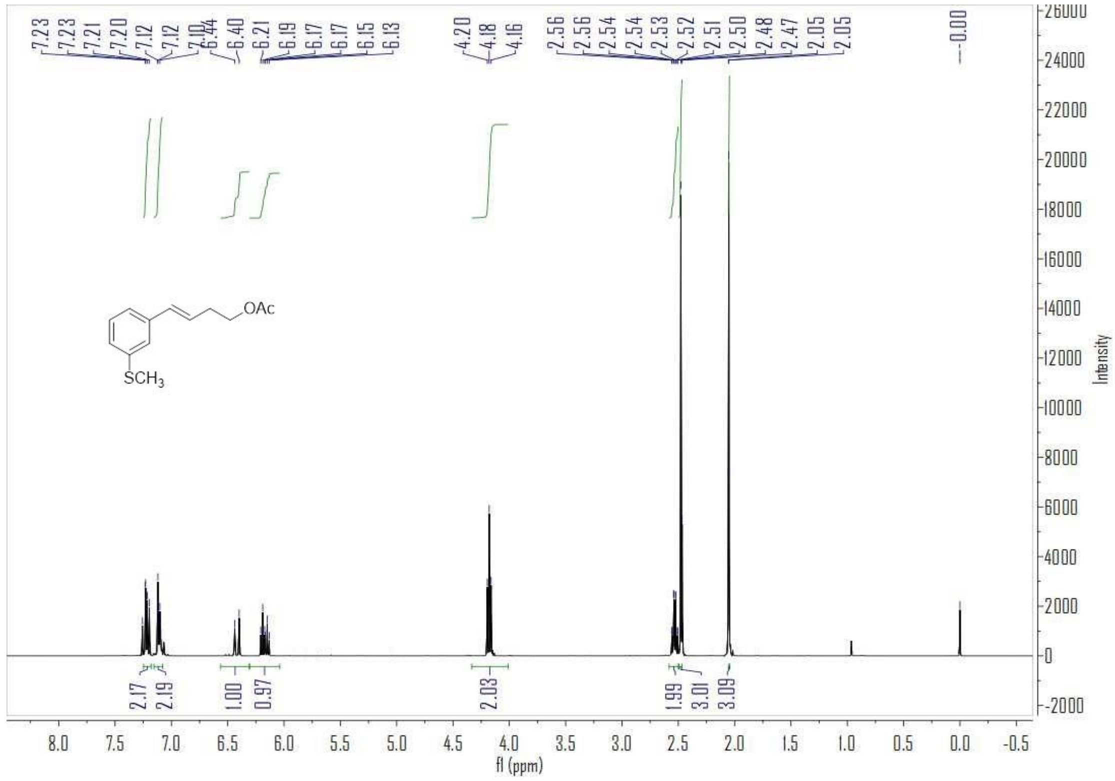

7.23
7.23
7.21
7.20
7.12
7.12
7.10
6.44
6.40
6.21
6.19
6.17
6.17
6.15
6.13
4.20
4.18
4.16
2.56
2.56
2.54
2.54
2.53
2.52
2.51
2.50
2.48
2.47
2.05
2.05
0.00
OAc
SCH_3
2.17
2.19
1.00
0.97
2.03
1.99
3.01
3.09
Intensity
fl (ppm)

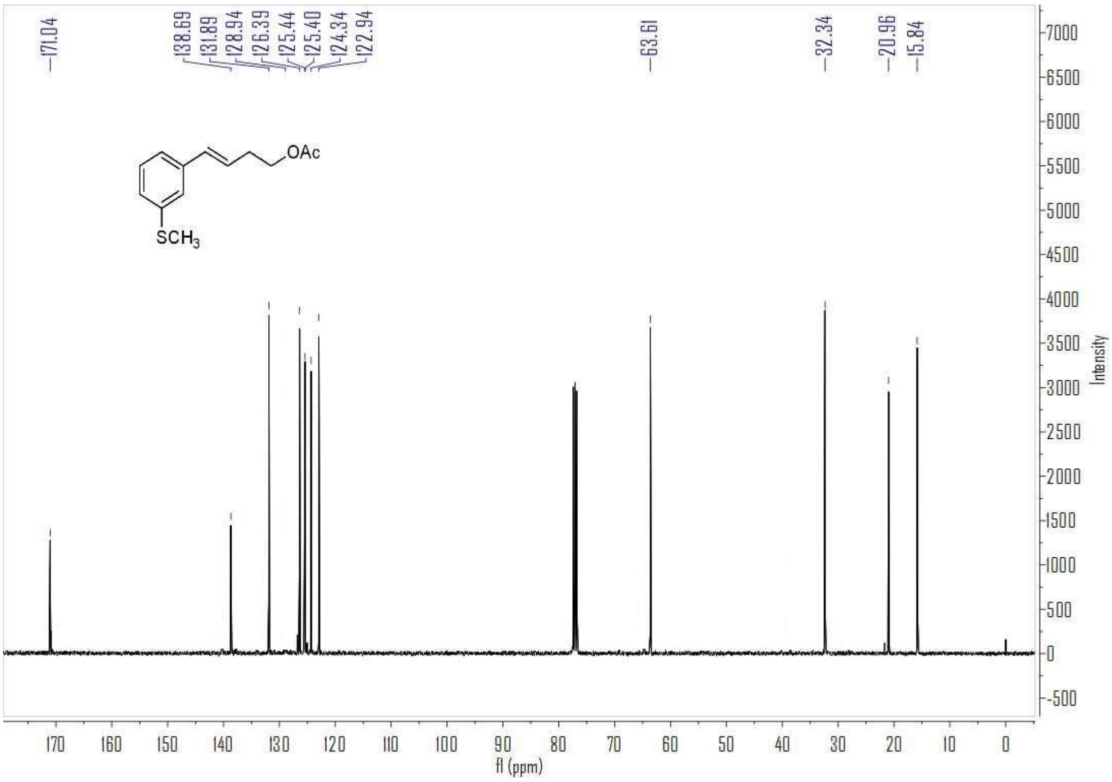
171.04
138.69
131.89
128.94
126.39
125.44
125.40
124.34
122.94
63.61
32.34
20.96
15.84
OAc
SCH3
Intensity
fl (ppm)

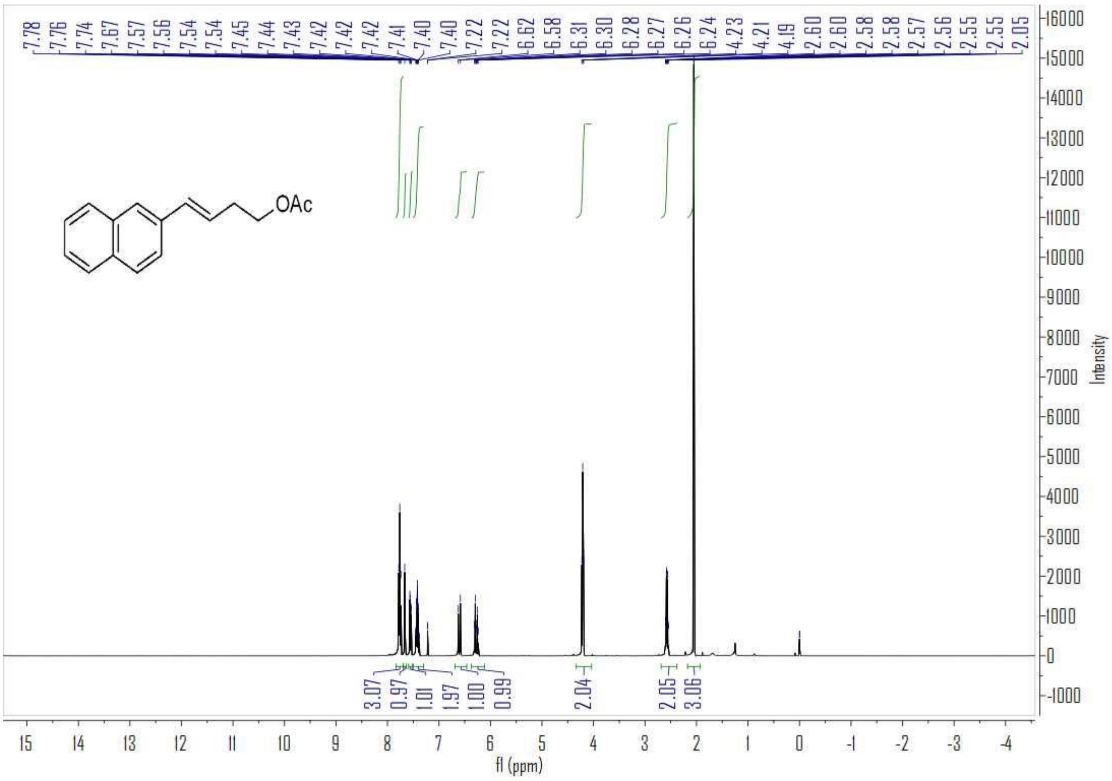
OAc
Intensity
3.07
0.97
1.01
1.97
1.00
0.99
2.04
2.05
3.06
fl (ppm)

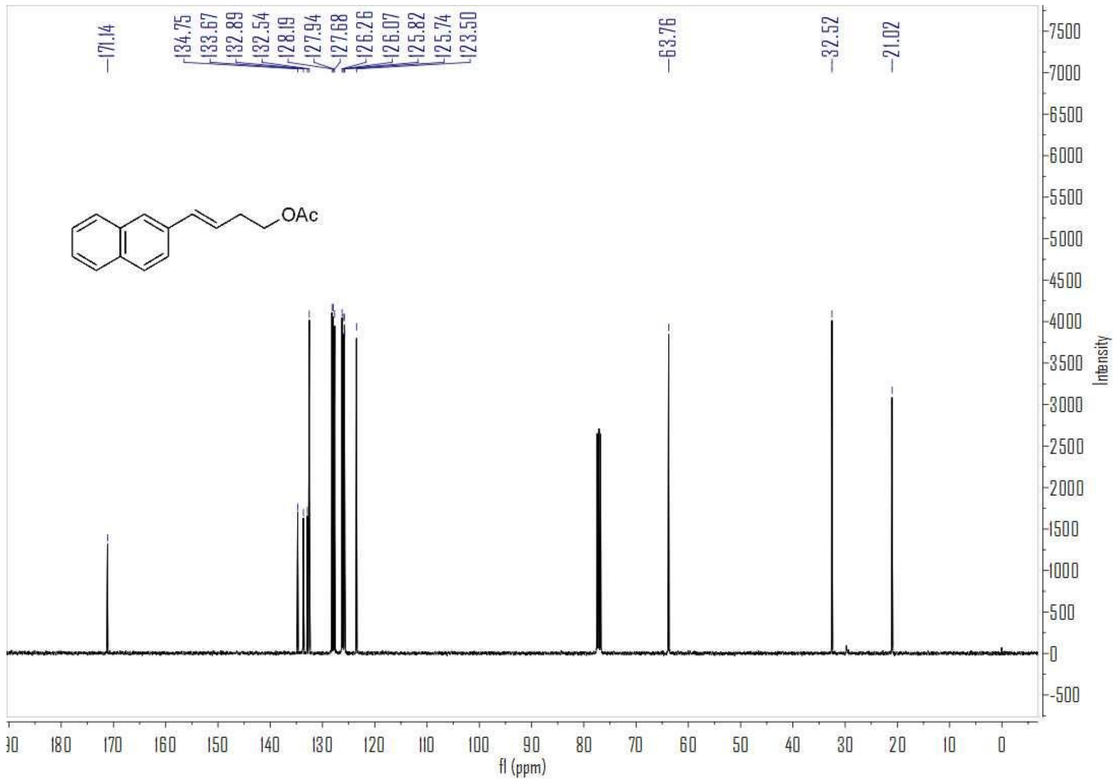

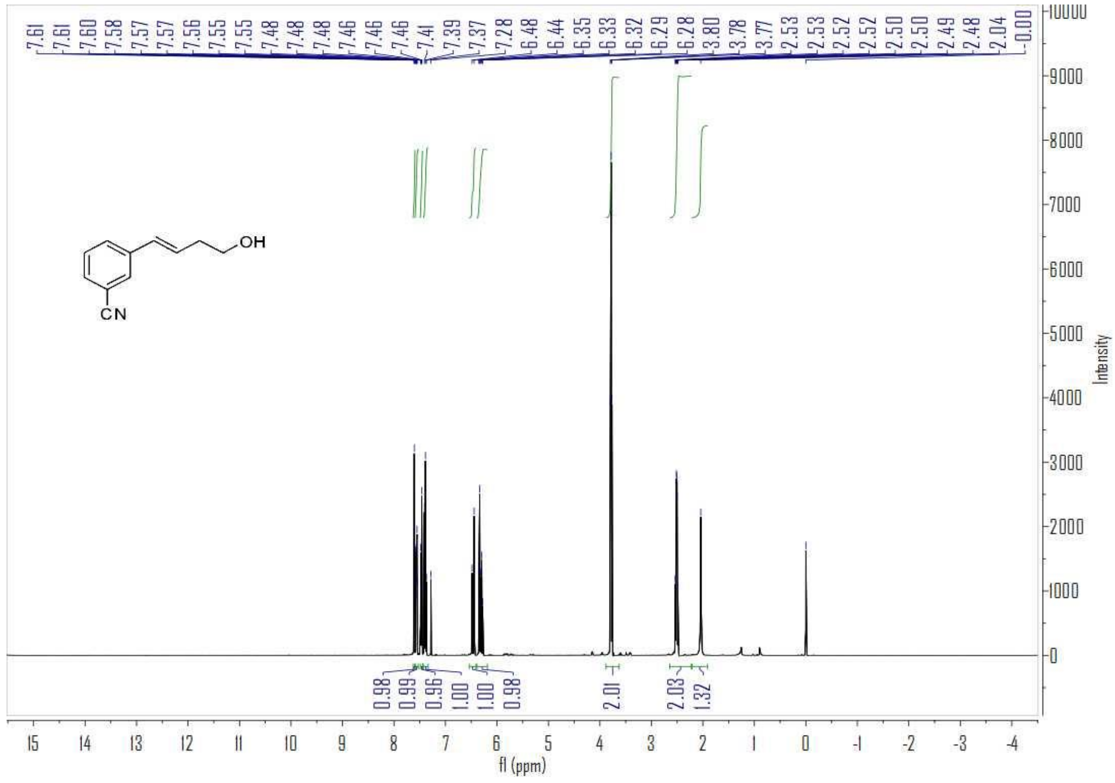

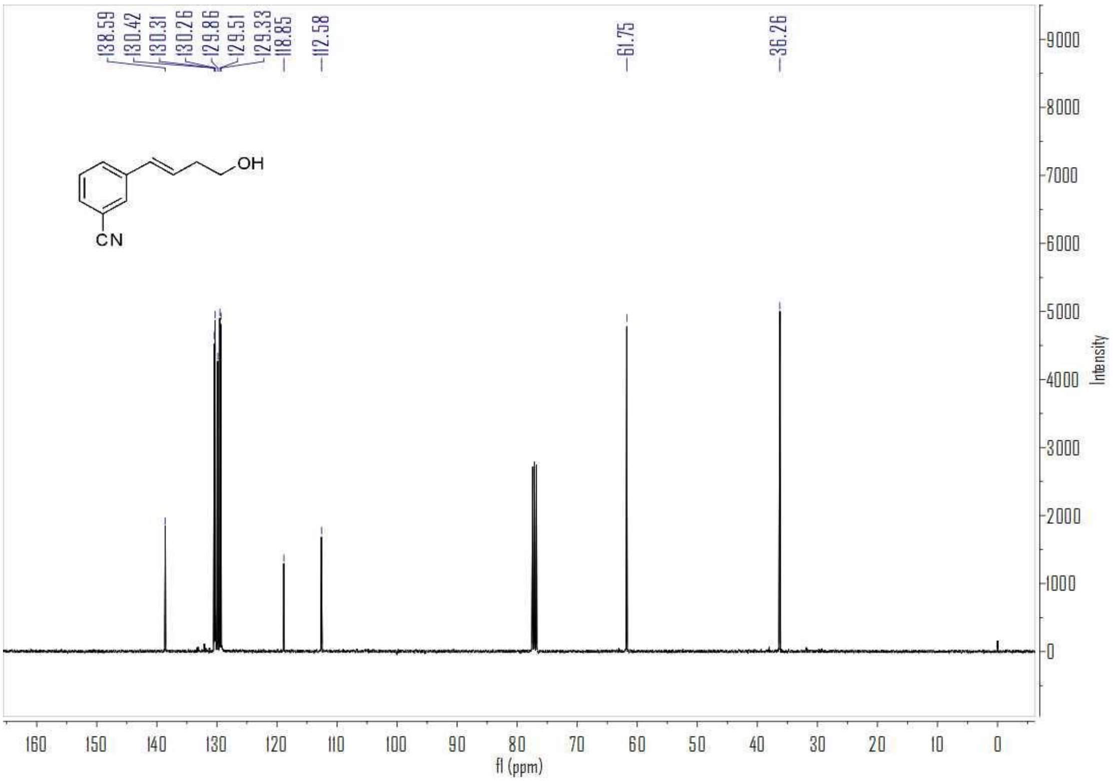
138.59
130.42
130.31
130.26
129.86
129.51
129.33
118.85
112.58
61.75
36.26
OH
CN
Intensity
f1 (ppm)

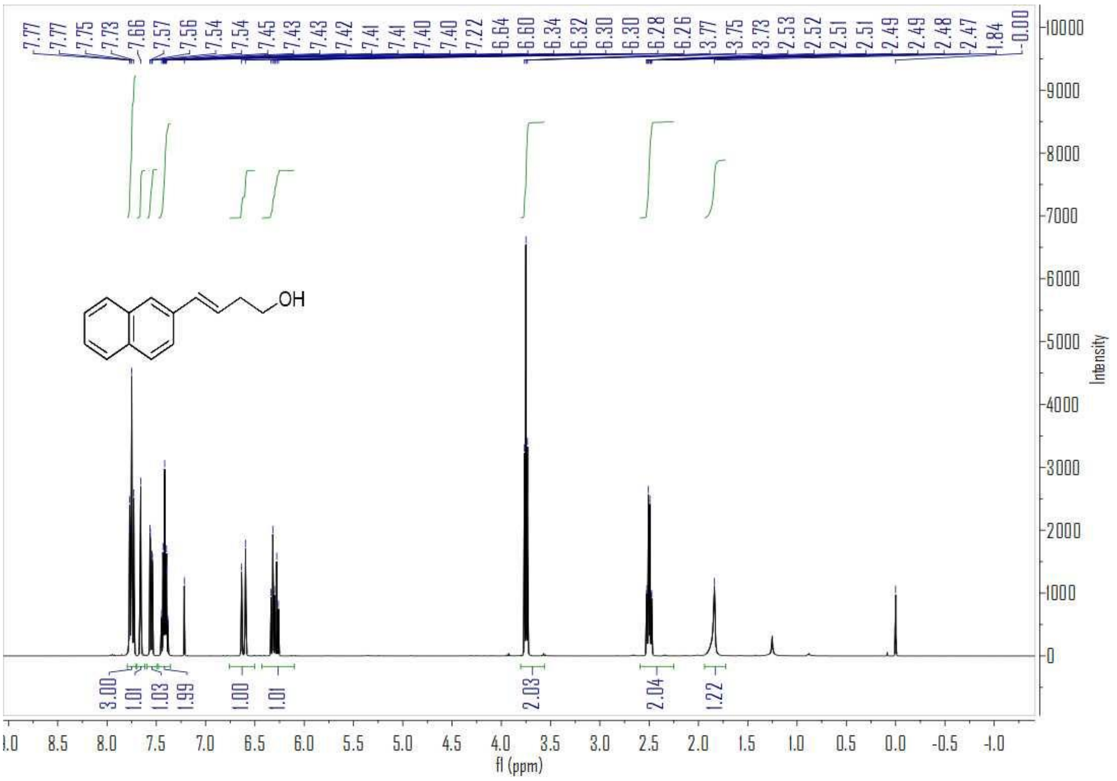
7.77
7.77
7.75
7.73
7.66
7.57
7.56
7.54
7.54
7.45
7.43
7.43
7.42
7.41
7.41
7.40
7.40
7.22
6.64
6.60
6.34
6.32
6.30
6.30
6.28
6.26
3.77
3.75
3.73
2.53
2.52
2.51
2.51
2.49
2.49
2.48
2.47
1.84
-0.00
OH
3.00
1.01
1.03
1.99
1.00
1.01
2.03
2.04
1.22
Intensity
f1 (ppm)

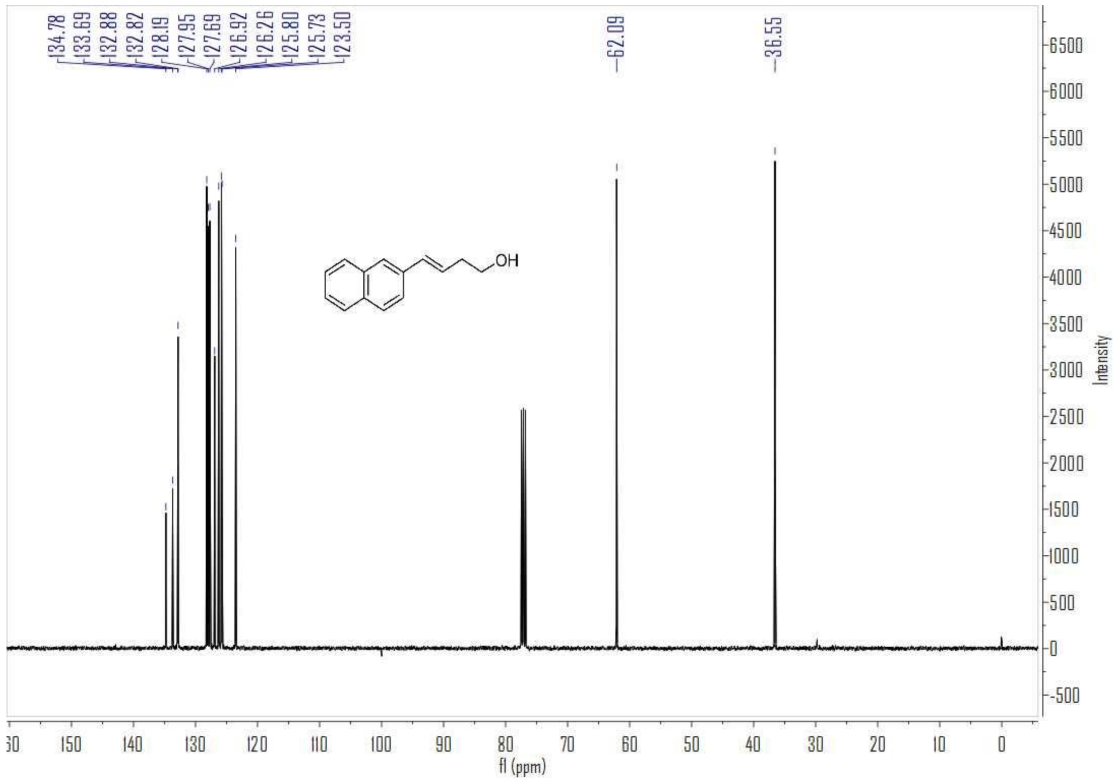

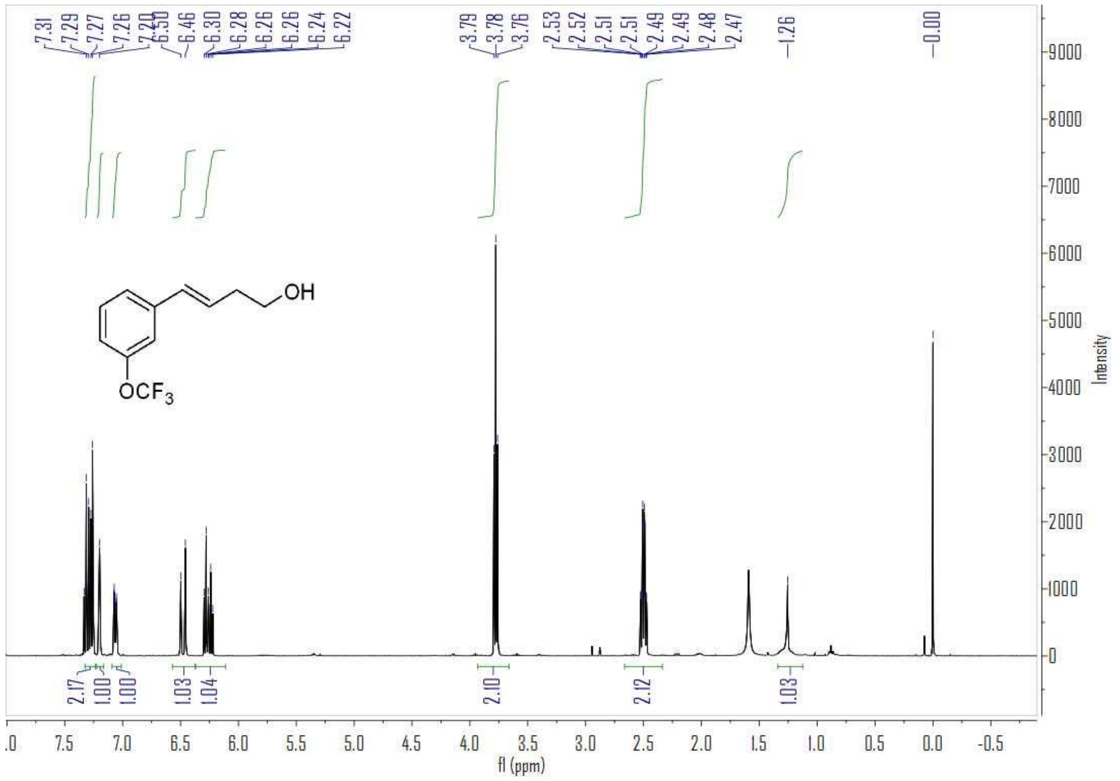

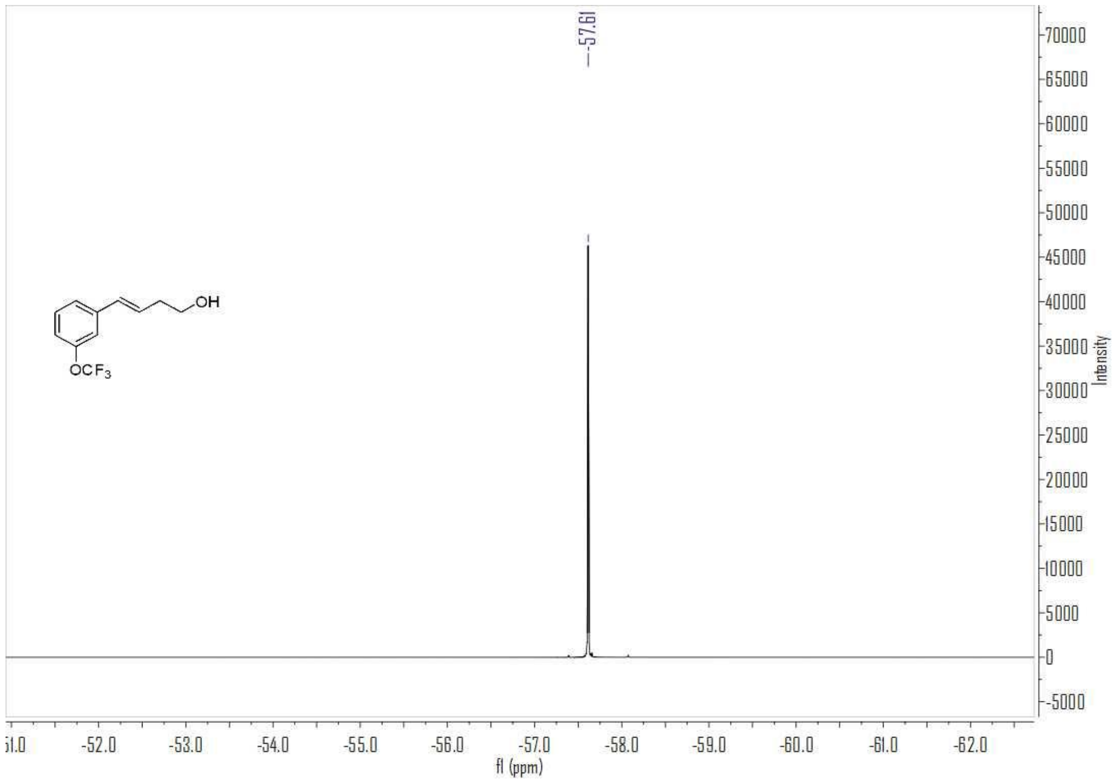

-57.61
OH
OCF3
Intensity
f1 (ppm)

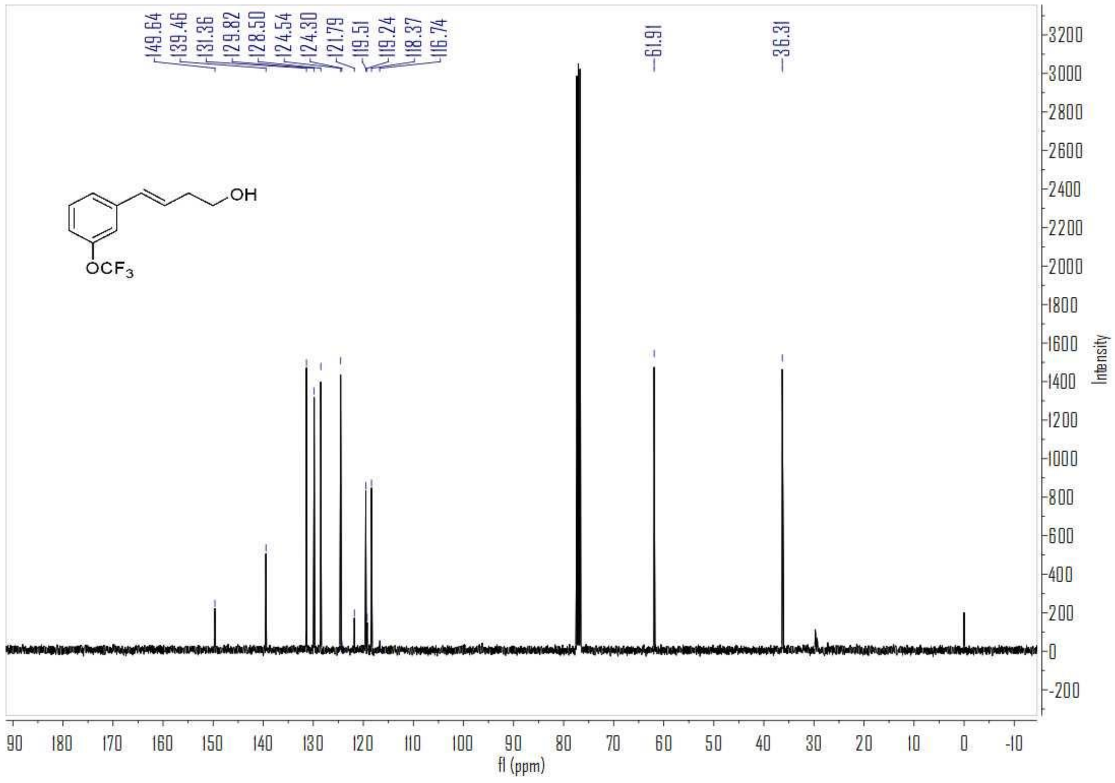

149.64
139.46
131.36
129.82
128.50
124.54
124.30
121.79
119.51
119.24
118.37
116.74
61.91
36.31
OH
OCF3
Intensity
f1 (ppm)

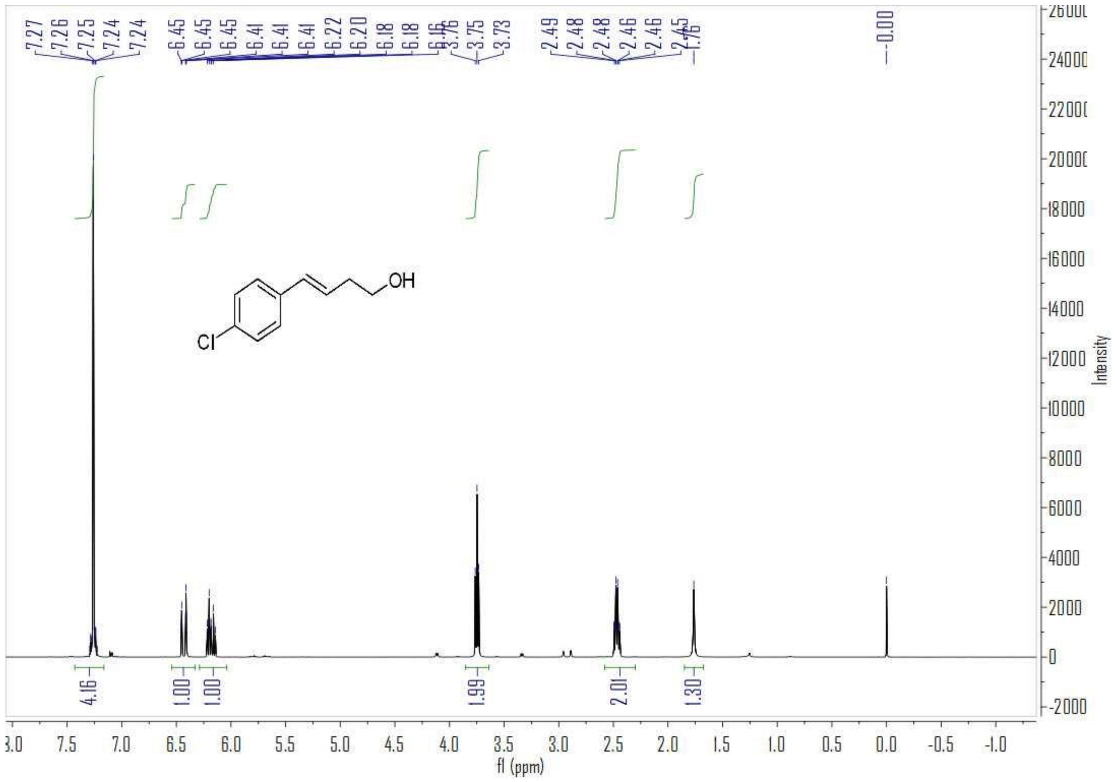

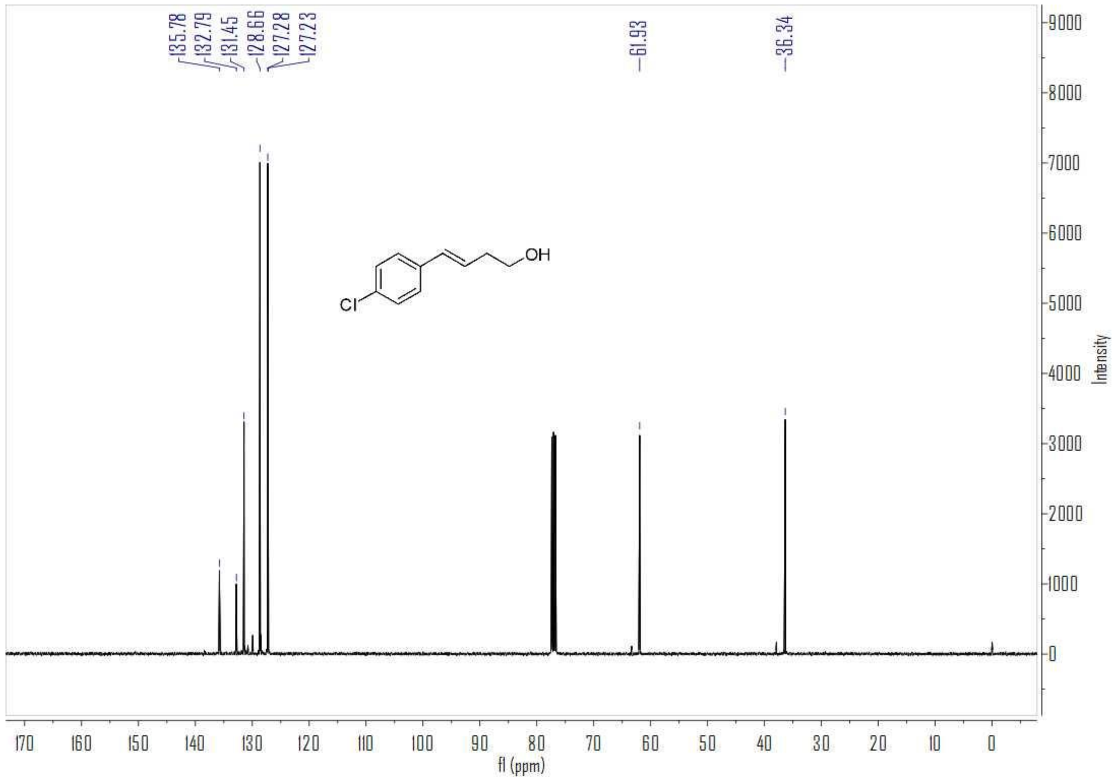

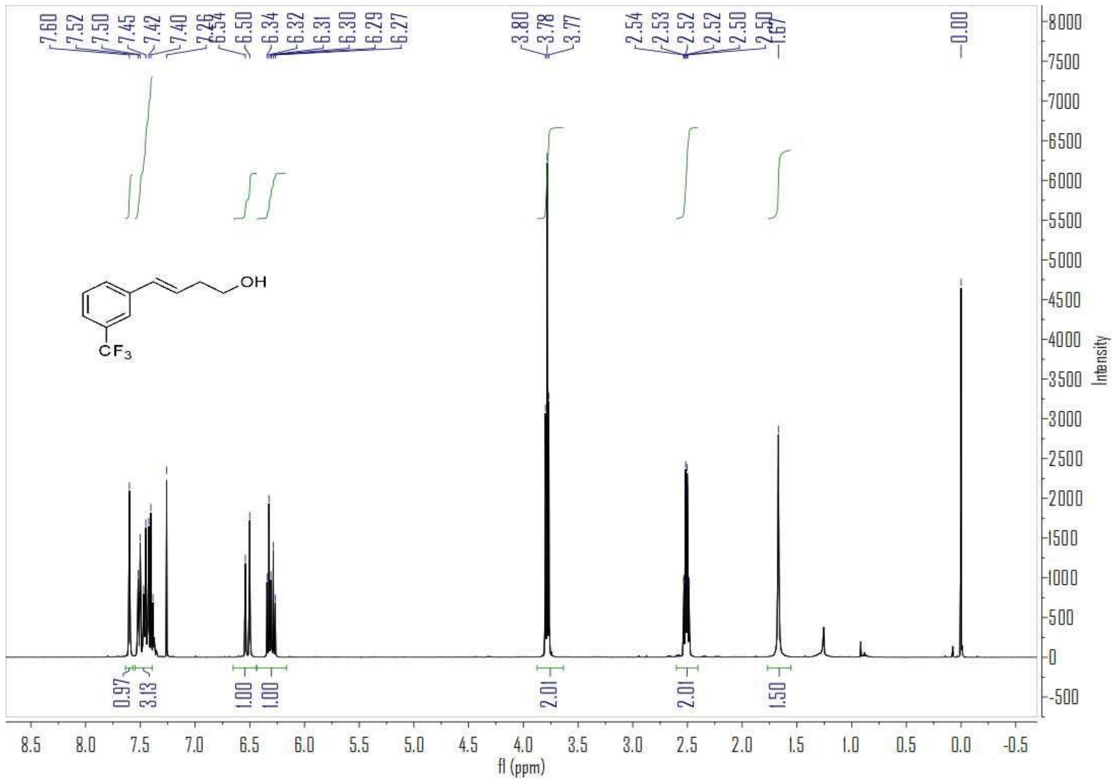

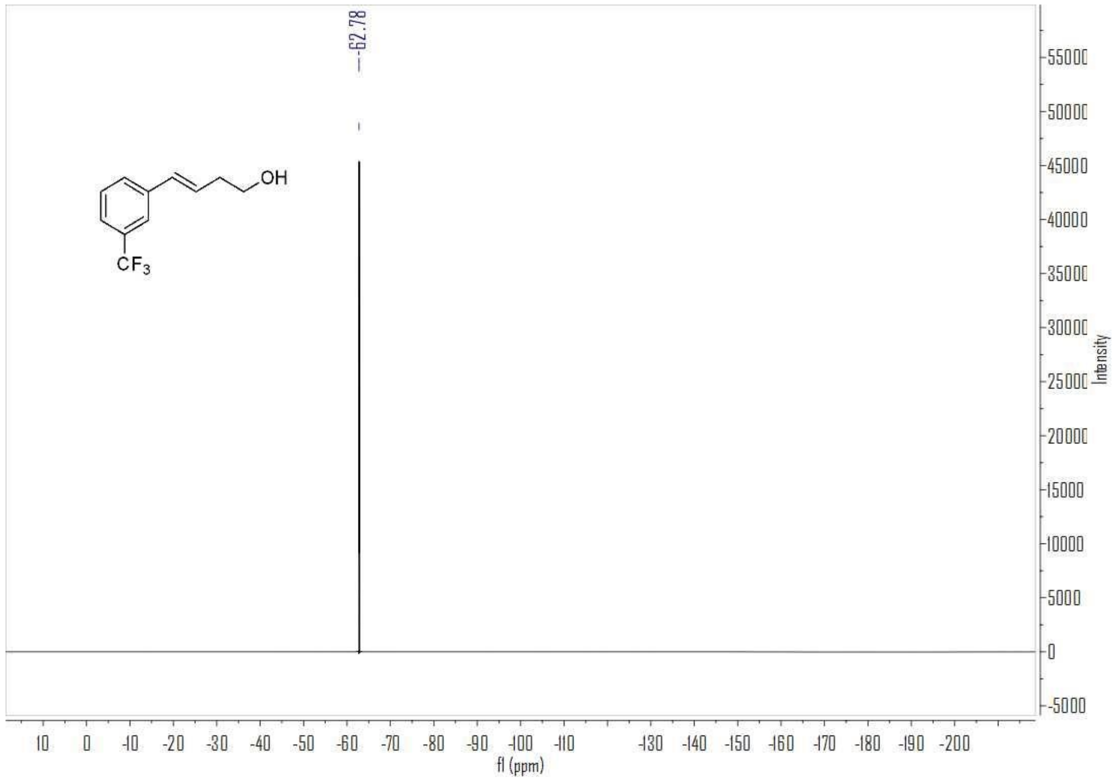

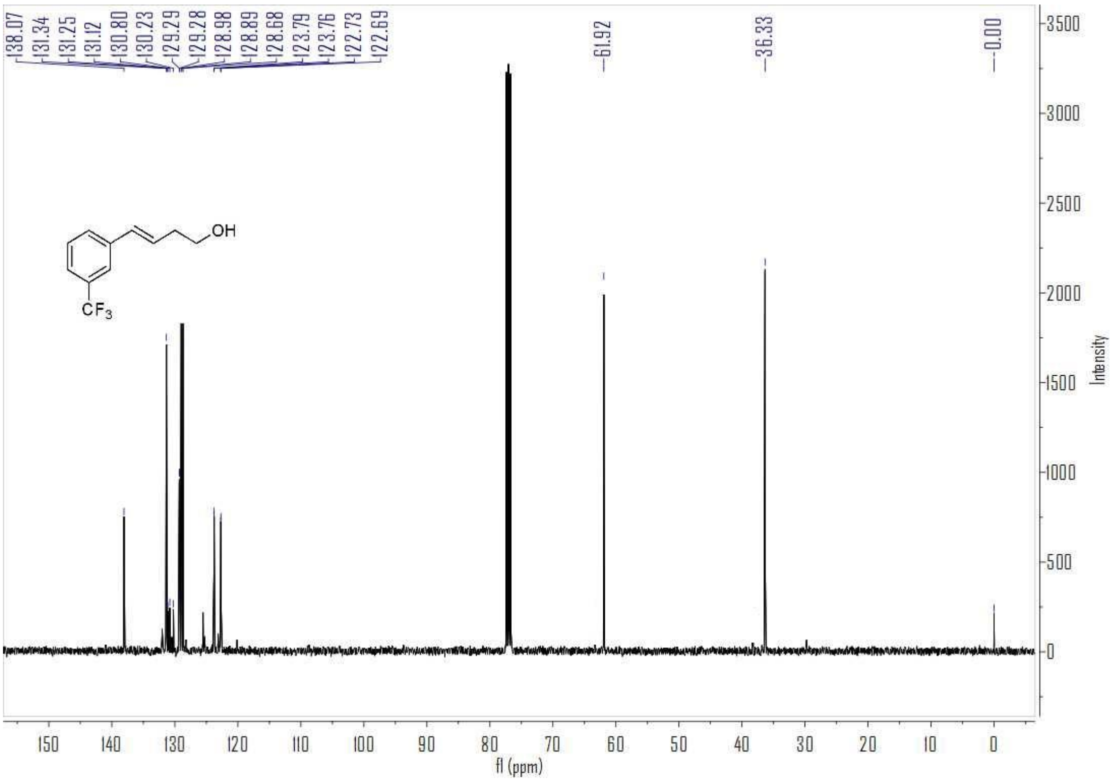

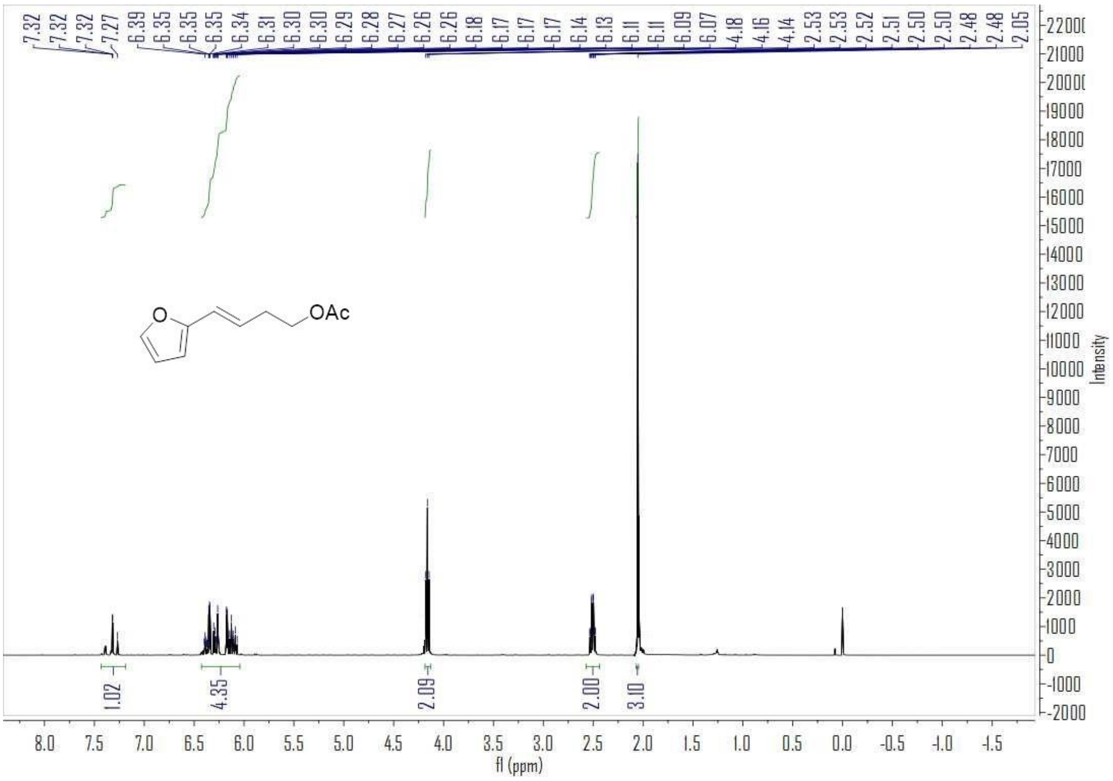

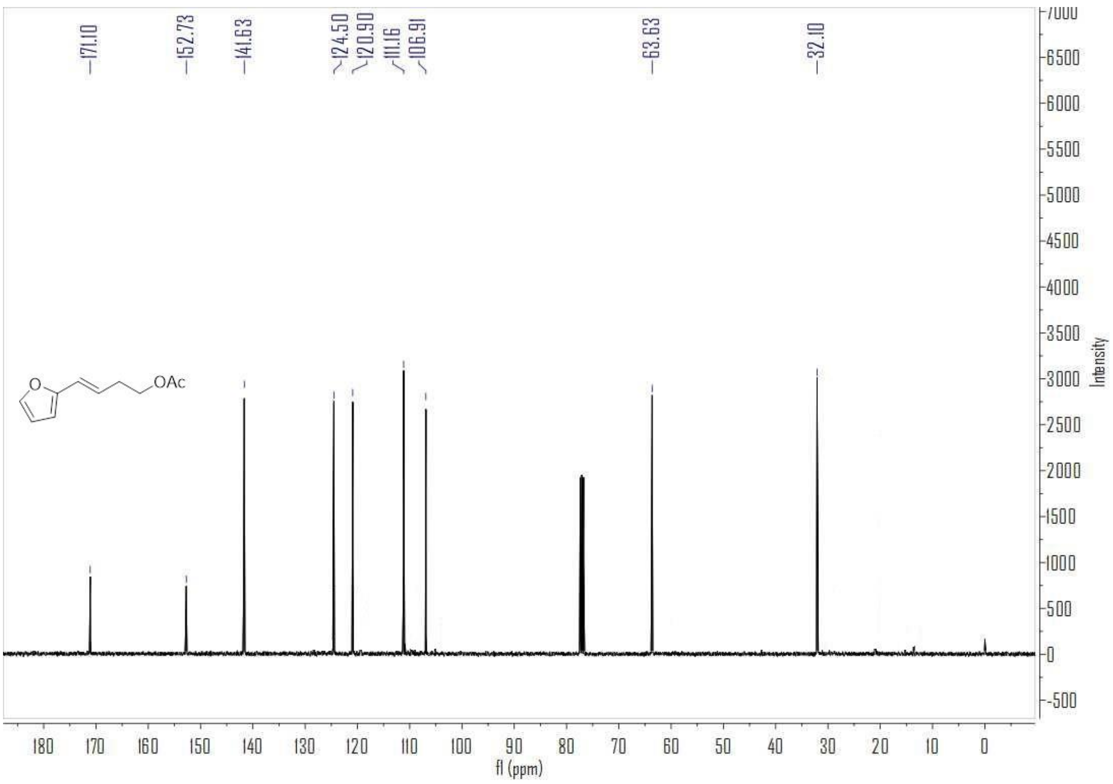

OAc
171.10
152.73
141.63
124.50
120.90
111.16
106.91
63.63
32.10
fl (ppm)
Intensity

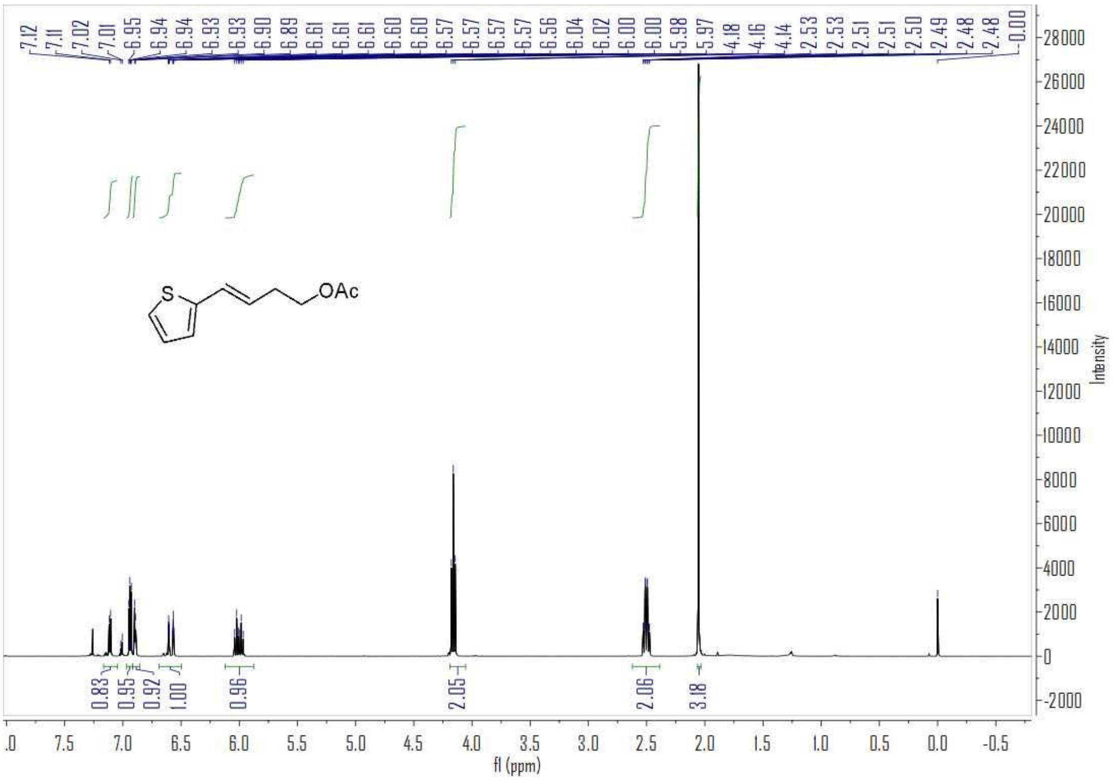

S
OAc
0.83
0.95
0.92
1.00
0.96
2.05
2.06
3.18
fl (ppm)
Intensity

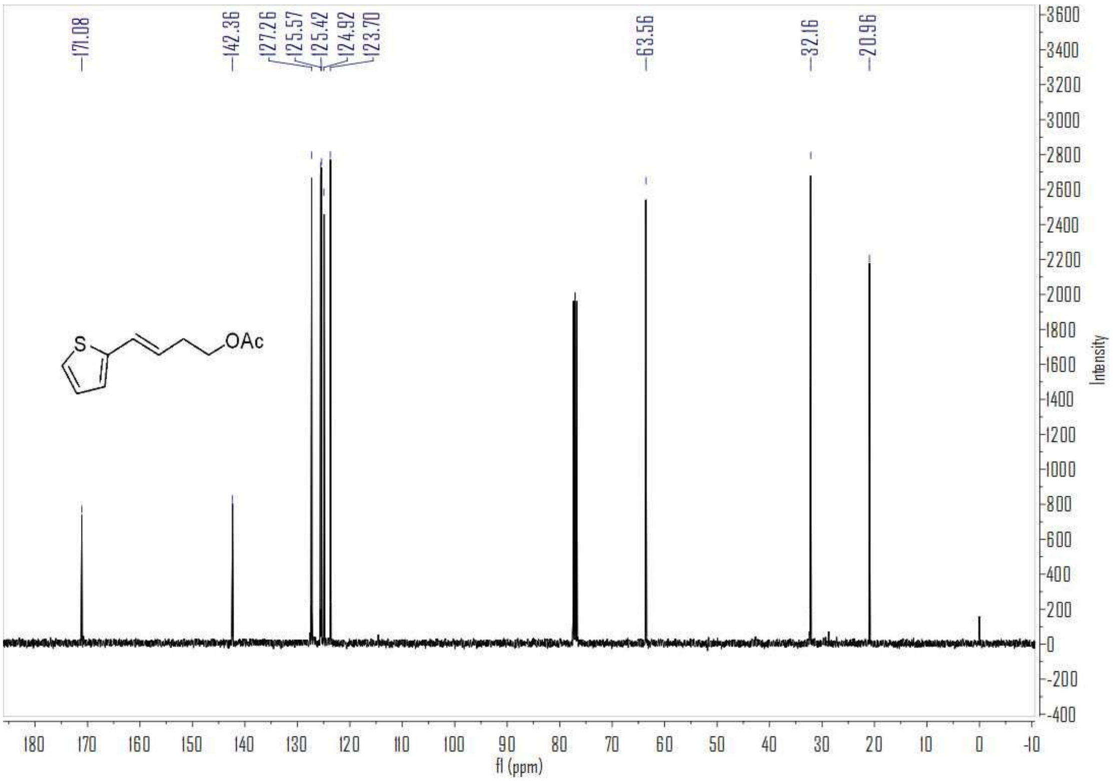
171.08
142.36
127.26
125.57
125.42
124.92
123.70
63.56
32.16
20.96
OAc
f1 (ppm)
Intensity

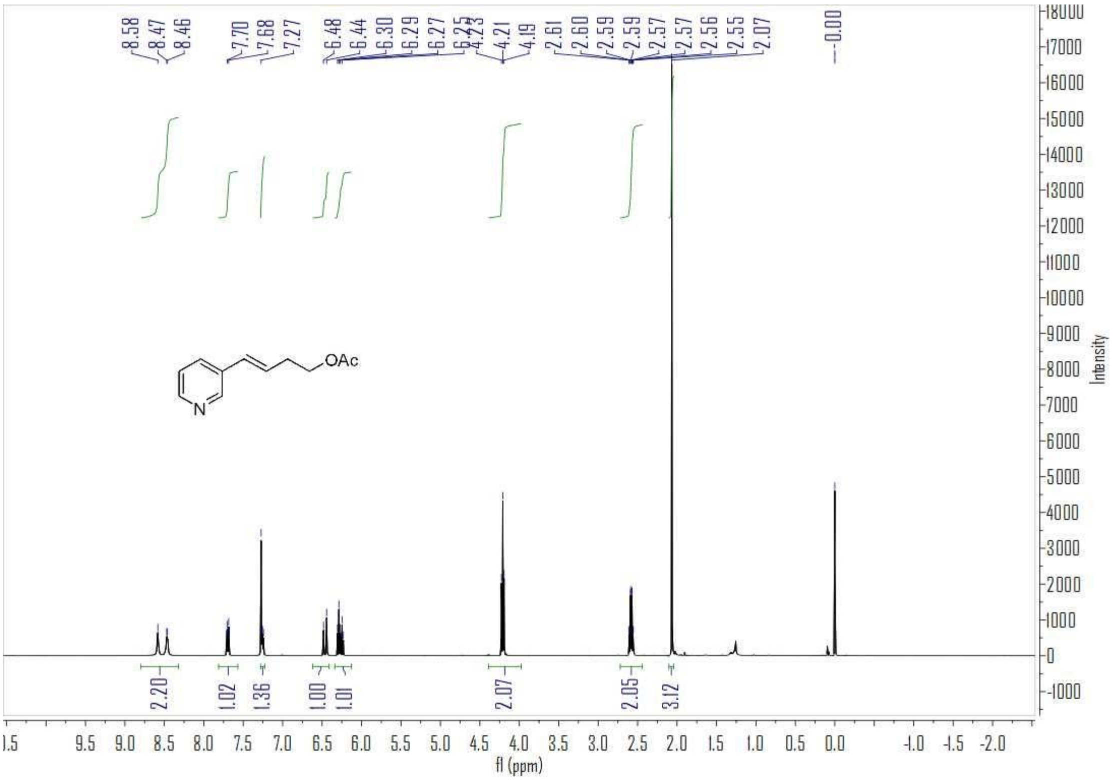
8.58
8.47
8.46
7.70
7.68
7.27
6.48
6.44
6.30
6.29
6.27
6.25
4.23
4.21
4.19
2.61
2.60
2.59
2.59
2.57
2.57
2.56
2.55
2.07
0.00
2.20
1.02
1.36
1.00
1.01
2.07
2.05
3.12
OAc
f1 (ppm)
Intensity

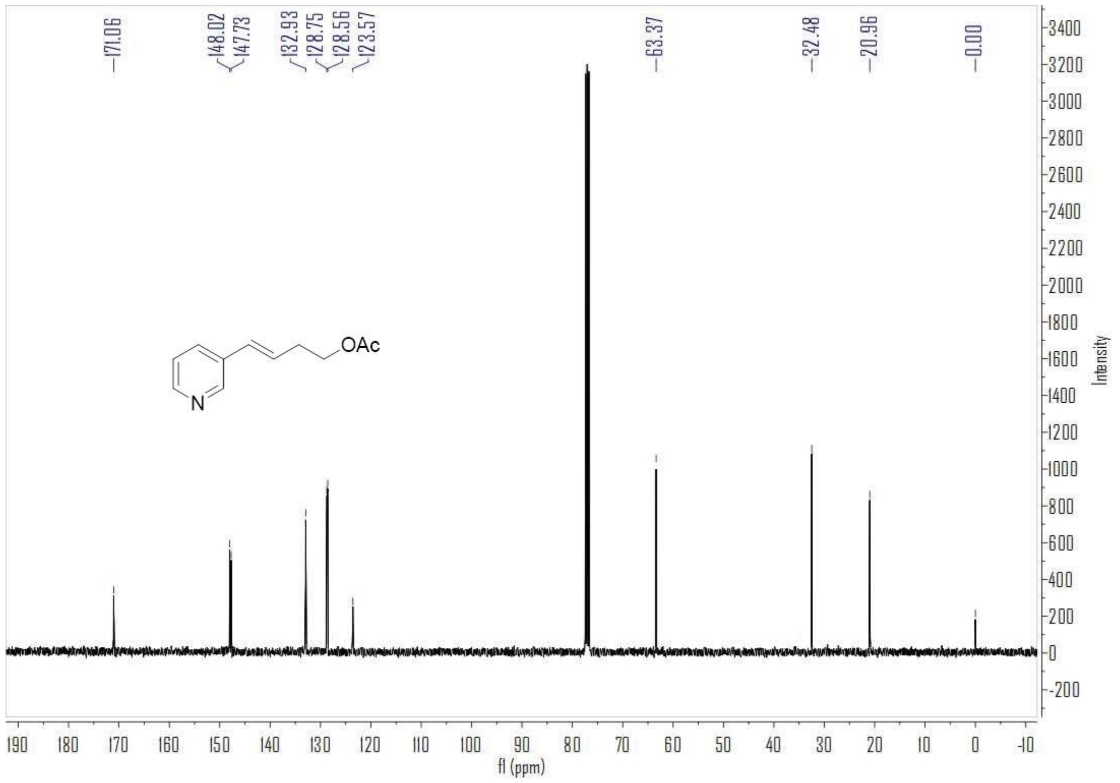

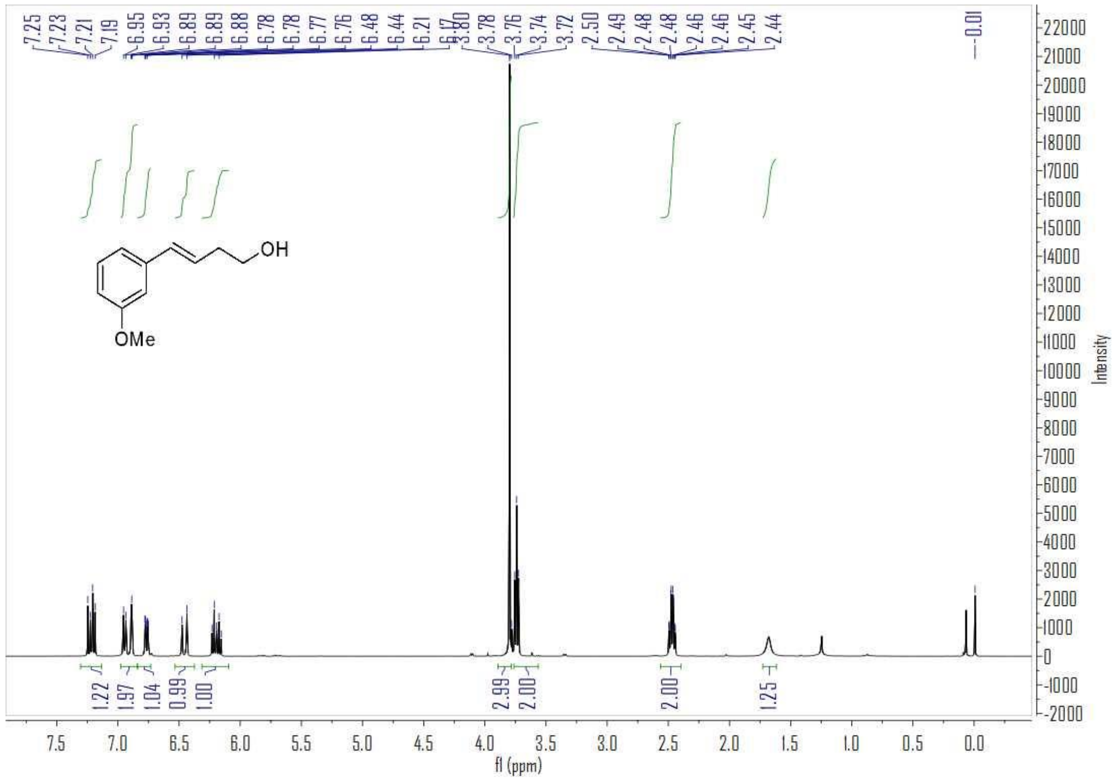

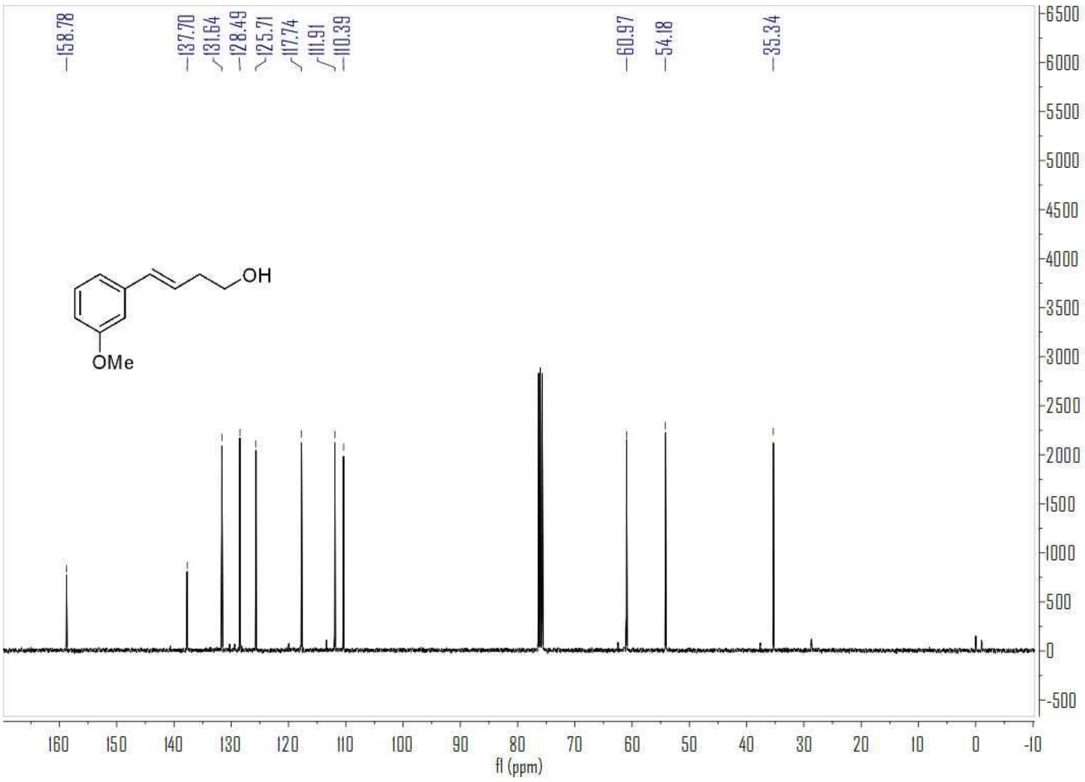

158.78
137.70
131.64
128.49
125.71
117.74
111.91
110.39
60.97
54.18
35.34
OH
OMe
Intensity
f1 (ppm)

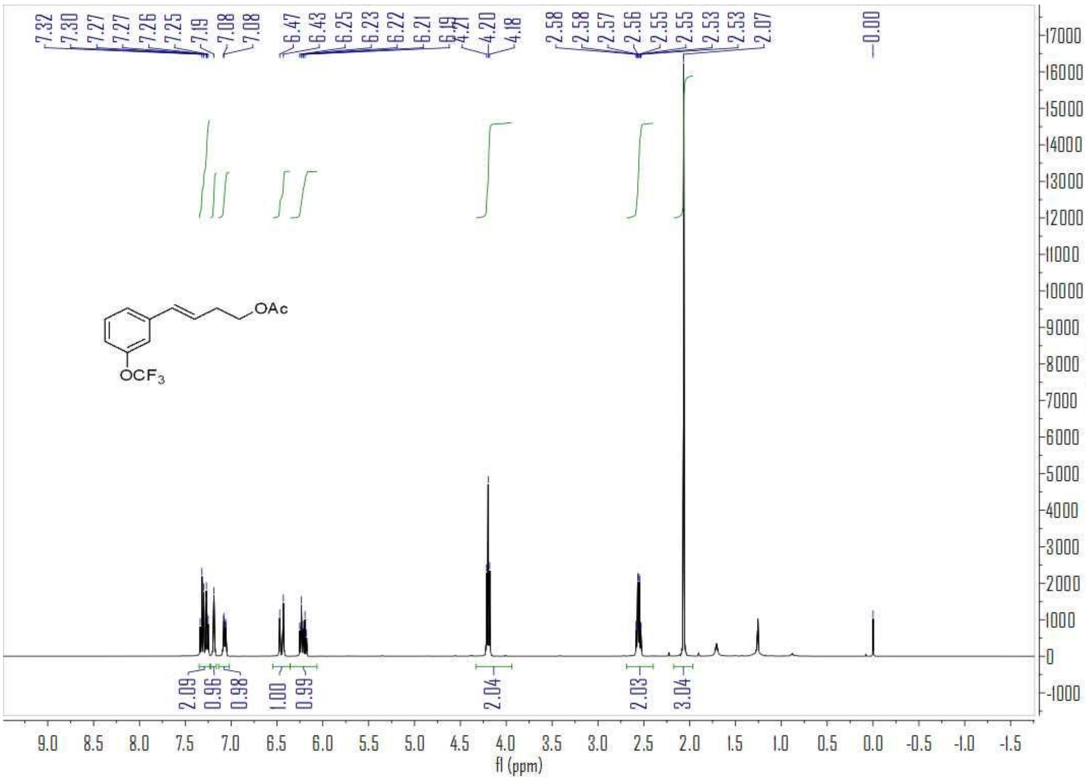

OAc
OCF3
2.09
0.96
0.98
1.00
0.99
2.04
2.03
3.04
0.00
Intensity
f1 (ppm)

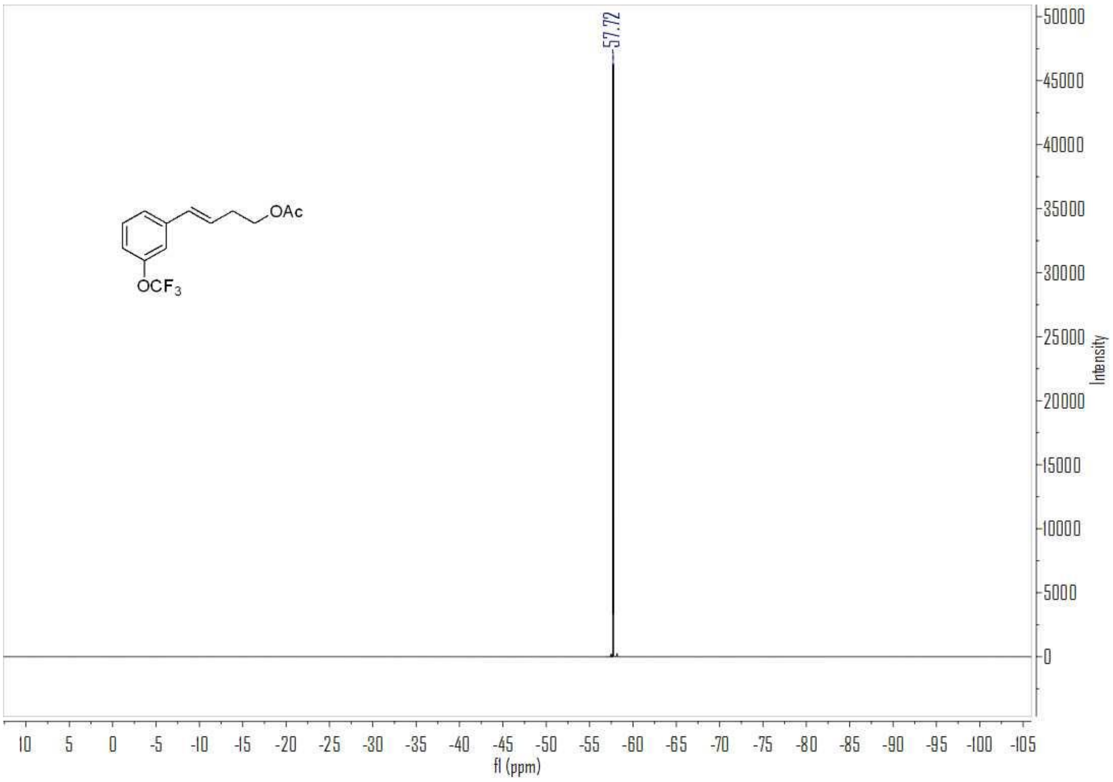
-57.72
OAc
OCF3
Intensity
fl (ppm)

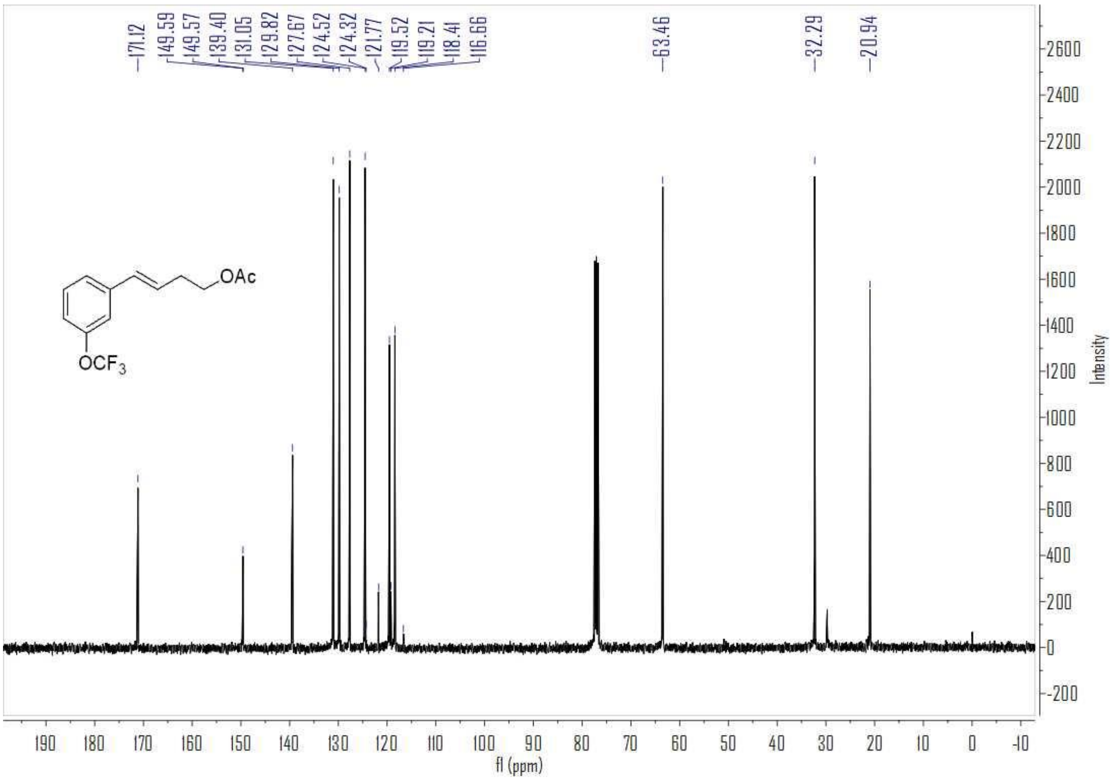
171.12
149.59
149.57
139.40
131.05
129.82
127.67
124.52
124.32
121.77
119.52
119.21
118.41
116.66
63.46
32.29
20.94
OAc
OCF3
Intensity
fl (ppm)
